# Developing a 5-Gene Signature Related to Pyroptosis for Osteosarcoma Patients

**DOI:** 10.1155/2022/1317990

**Published:** 2022-08-05

**Authors:** Zhe Li, Chi Jin, Xinchang Lu, Yan Zhang, Yi Zhang, Jia Wen, Yongkui Liu, Xiaoting Liu, Jiazhen Li

**Affiliations:** ^1^Orthopaedics, The First Affiliated Hospital of Zhengzhou University, Zhengzhou 410100, China; ^2^Orthopaedics, The Affiliated Hospital of Hangzhou Normal University, Hangzhou 310015, China

## Abstract

Although the incidence of osteosarcoma (OS) is relatively low compared with other cancer types, the overall survival of metastatic OS was less than 30%. This study aimed to reveal the role of pyroptosis in osteosarcoma and develop a prognostic model related to pyroptosis. Weighted correlation network analysis (WGCNA) was applied to identify key gene modules related to pyroptosis. Univariate Cox regression analysis was used to screen prognostic genes related to pyroptosis. The least absolute shrinkage and selection operator (LASSO) and stepwise Akaike information criterion (stepAIC) were employed to optimize and construct a prognostic model. Five prognostic genes (*COL13A1*, *TNFRSF1A*, *LILRA6*, *CTNNBIP1*, and *CD180*) related to pyroptosis were identified. According to the 5-gene signature, OS samples were divided into high- and low-PPRS groups with differential prognosis. Immune-related pathways were more activated in the low-PPRS group. The 5-gene signature was effective and robust to predict OS prognosis. These five prognostic genes were involved in OS development and may serve as new targets for developing therapeutic drugs.

## 1. Introduction

Sarcomas are a group of rare malignant tumors deriving from mesenchymal cells, with an incidence of about 2–4 per 100,000 [1]. Osteosarcoma (OS) is one of the types of sarcomas with an incidence of about 3.4 per million worldwide, which commonly occurs in children and adolescents [2]. Two peaks of its incidence rate are shown at the ages of 15–19 and 75–79 [3]. Osteosarcoma mostly localizes in long bones, especially in arms, legs, knees, and shoulders. Although localized osteosarcoma patients have a relatively high overall survival, reaching a 5-year survival of 70–75%, metastatic patients have no more than 30% largely due to the resistance to chemotherapy or radiotherapy [4].

Immunotherapy is developed as a potential therapy for cancers and has achieved satisfactory efficiency in some cancer types. Some immune checkpoint inhibitors such as antiprogrammed cell death protein 1 (PD-1) inhibitors have been approved by Food and Drug Administration (FDA) [5]. Evidence supports that immune checkpoint inhibitors can also be potential therapeutic drugs for osteosarcoma. Tawbi et al. observed that one of 22 OS patients had an objective response to an anti-PD-1 antibody [6]. However, in a phase 2 trial of pembrolizumab for treating advanced osteosarcoma, no significant antitumor activity of pembrolizumab was presented in 12 patients [7]. On the one hand, more effective immunotherapy is needed and on the other hand, reaching a more personalized therapy for patients is also important. That is, to identify patients who are more suitable or sensitive to immunotherapy is beneficial.

Currently, a number of prognostic signatures or molecular subtypes have been developed for different cancer types based on gene expression data. Pyroptosis, as an emerging hot spot in cancer, plays an important role in cancer cell proliferation and migration [8]. A number of studies reported that pyroptosis suppresses cancer cell growth in most the cancers such as glioma, ovarian cancer, gastric cancer, and colon cancer [8]. However, the tumor-promotive role of pyroptosis is shown in cervical cancer and esophageal adenocarcinoma [8]. The role of pyroptosis in osteosarcoma remains unclearly. Bioinformatics analysis helps a lot in exploring the mechanism of cancer development and the biomarkers for predicting cancer prognosis [9–11].

In this study, we revealed the relation between pyroptosis and osteosarcoma survival. By using weighted correlation network analysis (WGCNA) and Cox regression analysis, we identified key prognostic genes of osteosarcoma. A 5-gene signature related to pyroptosis was constructed, and its prognostic value was verified in three independent datasets. In addition, we evaluated the relation between the signature and tumor microenvironment (TME). The 5-gene signature was demonstrated to have an ability to identify individuals who were more sensitive to immunotherapy.

## 2. Materials and Methods

### 2.1. Data Information

The flow chart of this study is shown in Supplementary Figure S1. The TARGET-OS dataset containing RNA-seq data was downloaded from National Cancer Institute Genomic Data Commons Data Portal (https://portal.gdc.cancer.gov/). GSE21257 and GSE39055 datasets containing expression data of osteosarcoma samples were downloaded from Gene Expression Omnibus (GEO) database (https://www.ncbi.nlm.nih.gov/geo/). For the TARGET-OS dataset, samples without survival time and status were removed. Ensembl ID was converted to gene symbol by using hgu133plus2. db *R* package. The median expression value of one gene was selected when the gene had multiple gene symbols. For GSE cohorts, probes were matched to gene symbols by using hgu133plus2. db *R* package. The median expression value of one gene was selected when the gene matched multiple probes. One probe was excluded when they had multiple genes. Finally, 86, 53, and 37 osteosarcoma samples remained in TARGET-OS, GSE21257, and GSE39055 datasets, respectively.

### 2.2. Identifying Key Genes Related to Prognosis and Pyroptosis

A gene set of “REACTOME_PYROPTOSIS” was downloaded from Molecular Signature Database (MSigDB, https://www.gsea-msigdb.org/gsea/msigdb/). Single sample gene set enrichment analysis (ssGSEA) in GSVA *R* package [12] was performed to calculate ssGSEA score of pyroptosis based on “REACTOME_PYROPTOSIS” gene set in the TARGET-OS dataset. Samples were divided into two groups with high and low scores of pyroptosis according to the median value. WGCNA [13] was applied to identify key gene modules related to pyroptosis. Firstly, samples were clustered to screen the coexpression network. To meet the standard of a scale-free network, a correlation coefficient >0.85 was determined. Then, the expression matrix was transferred to the topology matrix. Based on the topological overlap matrix, average-linkage clustering was used to cluster genes with each gene module containing at least 30 genes. Next, eigengenes of each gene module were calculated, and modules were further clustered and combined with conditions of height = 0.25, deepSplit = 2, and minModuleSize = 30. The correlation between each module and pyroptosis score was assessed. The most significant gene module was chosen to be the key module and used to construct a prognostic model.

### 2.3. Constructing a Prognostic Model Based on the Key Gene Module

Univariate Cox regression analysis was performed on genes within the purple gene module for screening prognosis-related genes in the TARGET-OS dataset (*P* < 0.05). The least absolute shrinkage and selection operator (LASSO) Cox regression analysis in glmnet *R* package [14] was used to decrease the number of prognostic genes by constructing a penalty function-based model. The coefficients of each gene were compressed with changing lambda values. Coefficients closed to zero with the increasing lambda values. 5-fold cross-validation was used to validate the model. Stepwise Akaike information criterion (stepAIC) in MASS *R* package [15] was further introduced to decrease the number of prognostic genes. Finally, the prognostic model was established as follows: pyroptosis-related prognostic risk score (PPRS) = coefficient 1^*∗*^gene 1+ coefficient 2^*∗*^gene 2+,… + coefficient *n*^*∗*^gene *n*.

### 2.4. Validating the Prognostic Model

PPRS was calculated for each sample in the TARGET-OS dataset. Samples were divided into high-PPRS and low-PPRS groups according to the optimal cut-off determined by survminer *R* package (http://www.sthda.com/english/rpkgs/survminer/). Receiver operating characteristic (ROC) analysis in the timeROC *R* package [16] was used to evaluate the effectiveness of the prognostic model to predict 1-year, 3-year, and 5-year overall survival. The area under ROC curve (AUC) was calculated. Kaplan–Meier survival analysis was performed to assess overall survival between high- and low-PPRS groups. By using the same methods, we validated the model in GSE21257 and GSE39055 datasets.

### 2.5. Gene Set Enrichment Analysis (GSEA)

GSEA is a popular methodology that allows calculation of the enrichment score based on the expression of a gene set [17]. SsGSEA is an extended methodology based on GSEA, which enables the calculation of the enrichment score for each sample [18]. We used ssGSEA to evaluate the enrichment of the pyroptosis pathway and hallmark pathways. ClusterProfiler *R* package was applied to annotate Kyoto Encyclopedia of Genes and Genomes (KEGG) pathways and gene ontology (GO) terms [19]. The top 10 significantly enriched pathways and GO terms were visualized (*P* < 0.05).

### 2.6. Evaluation of Tumor Microenvironment

CIBERSORT (http://cibersort.stanford.edu/) was employed to assess the estimated proportion of 22 immune cells in high- and low-PPRS groups [20]. Estimation of STromal and Immune cells in MAlignant Tumours using Expression data (ESTIMATE) was applied to calculate the stromal score, immune score, and ESTIMATE score [21]. Immune checkpoints obtained from HisgAtlas database were analyzed [22]. Tumor Immune Dysfunction and Exclusion (TIDE, http://tide.dfci.harvard.edu/) was implemented to predict the response of high- and low-PPRS groups to immunotherapy [23]. A higher TIDE score represents a higher immune escape from immunotherapy.

### 2.7. Statistical Analysis

All statistical analysis was conducted in *R* software (v4.1.1). Parameters of methodologies not indicating were default. The log-rank test was conducted in Kaplan–Meier survival analysis and univariate and multivariate Cox regression analysis. The Wilcoxon test was conducted to test the difference between the two groups. The Kruskal–Walls test was performed among four groups. *P* < 0.05 was considered significant (Ns, not significant. ^*∗*^*P* < 0.05, ^*∗∗*^*P* < 0.01, ^*∗∗∗*^*P* < 0.001, and ^*∗∗∗∗*^*P* < 0.0001).

## 3. Results

### 3.1. Pyroptosis Is Associated with Overall Survival of OS

**Figure 1:**
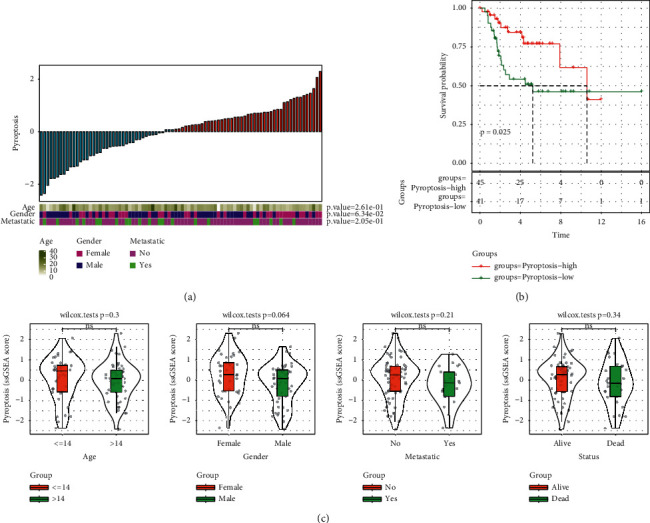
The relation between pyroptosis and clinical features of osteosarcoma in TARGET-OS dataset. (a) The distribution of different clinical features ranked by the z-score of ssGSEA score of pyroptosis. (b) Kaplan–Meier survival analysis of high- and low-score groups according to the cut-off of z-score = 0. The log-rank test was conducted. (c) The distribution of pyroptosis scores in different clinical features. The Wilcoxon test was conducted. ns, no significance.

To understand the relation between pyroptosis and OS prognosis, we calculated the ssGSEA score of pyroptosis for each sample in the TARGET-OS dataset. We found that the distribution of clinical features including age, gender, and metastasis was significantly associated with the enrichment score of pyroptosis (*P* < 0.0001, Figure 1(a)). Samples were divided into two groups with high and low ssGSEA scores. Kaplan–Meier survival analysis showed that two groups had differential overall survival (Figure 1(b)), suggesting that pyroptosis played an important role in tumor progression. However, no significant difference was observed between different groups of clinical features including age, gender, metastasis, and survival status (Figure 1(c)).

### 3.2. Identifying Key Genes Related to Pyroptosis by WGCNA

**Figure 2:**
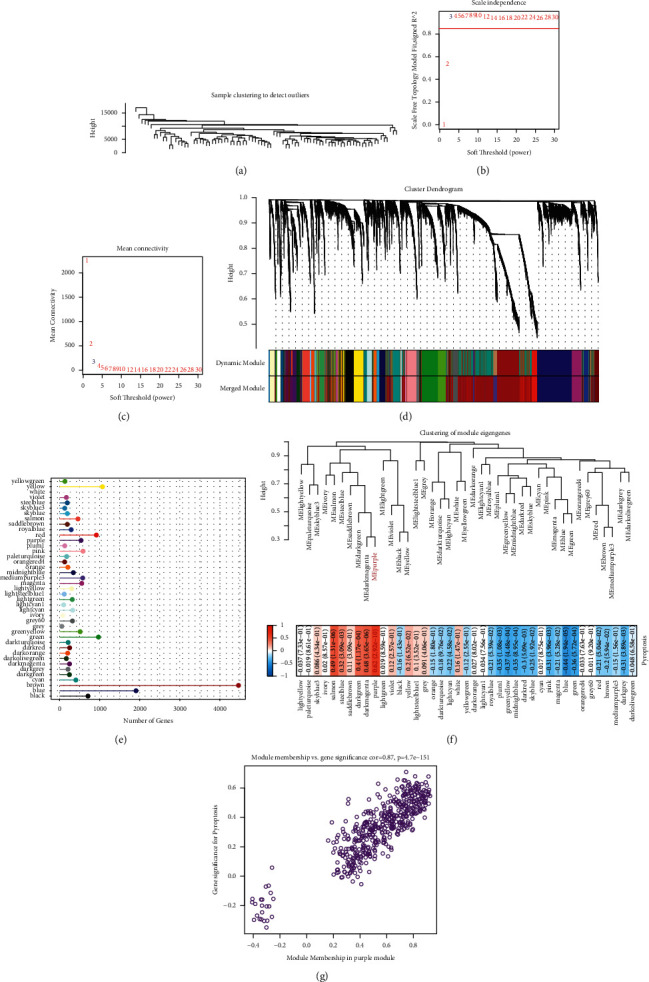
WGCNA for identifying key gene modules related to pyroptosis. (a) Clustering for TARGET-OS samples. (b, c) Analysis of scale independence and mean connectivity for different soft thresholds (power). (d) Cluster dendrogram based on topology and identification of gene modules. (e) Gene numbers of each gene module. (f) Pearson correlation analysis between gene modules and pyroptosis. Correlation coefficients were indicated by colors. Blue indicates negative correlation, and red indicates positive correlation. (g) Pearson correlation analysis between pyroptosis score (gene significance) and gene expression in the purple module (module membership).

We confirmed that the pyroptosis score was significantly associated with OS prognosis. To identify key genes related to pyroptosis, we applied WGCNA to cluster samples and screen co-expression modules (Figure 2). Samples in the TARGET-OS dataset were clustered (Figure 2(a)). To reach a scale-free network, a negative correlation over 0.85 between log (*k*) and log (P(k)) was selected, where *k* indicated connectivity degree. Therefore, the power of soft threshold (*β*) = 3 was confirmed (Figures 2(b) and 2(c)). By using average linkage clustering based on the topological overlap matrix (TOM), samples were further clustered. According to the dynamic cut method, modules were primarily distributed with each module containing at least 30 genes (Figure 2(d)). Then, adjacent modules were merged based on the eigengenes of each module, and finally, 41 modules remained (Figure 2(d)). The gene counts of each module were visualized (Figure 2(e)). Furthermore, we assessed the correlation between each module and pyroptosis. As a result, we observed that the purple module was significantly correlated with pyroptosis (*R* = 0.62, *P*=2.92*e* − 10, Figure 2(f)). In addition, the expression of genes in the purple module was positively correlated with the enrichment of pyroptosis (*R* = 0.87, *P*=4.7*e* − 151, Figure 2(g)). Therefore, the purple module was considered as a pyroptosis-related module for the following analysis. Functional analysis of KEGG pathways and GO terms for genes within the purple module showed that some immune-related terms were significantly enriched, such as neutrophil activation involved in immune response, MHC protein complex, immunoglobulin binding, and MHC protein complex binding (Supplementary Figure S2).

### 3.3. Constructing a Prognostic Model Based on Pyroptosis-Related Genes

Next, we utilized univariate Cox regression analysis and screened 187 prognostic genes within pyroptosis-related genes in the TARGET-OS dataset, with 10 positively (risk) correlating with prognosis and 177 negatively (protective) correlating with prognosis (*P* < 0.05, Supplementary Figure S3A). To construct a model using the minimum prognostic genes, we introduced LASSO Cox regression and stepAIC to reduce the number of prognostic genes. In LASSO analysis, the coefficients of prognostic genes were close to zero with the increasing lambda value (Supplementary Figure S3B). 5-Fold cross-validation revealed the confidence interval for each lambda value (Supplementary Figure S3C). When lambda = 0.1395, the model was optimal. Moreover, stepAIC was conducted to further optimize the model. Finally, 5 prognostic genes remained, with one risk gene (*COL13A1*) and four protective genes (*TNFRSF1A*, *LILRA6*, *CTNNBIP1*, and *CD180*) (Supplementary Figure S3D).

**Figure 3:**
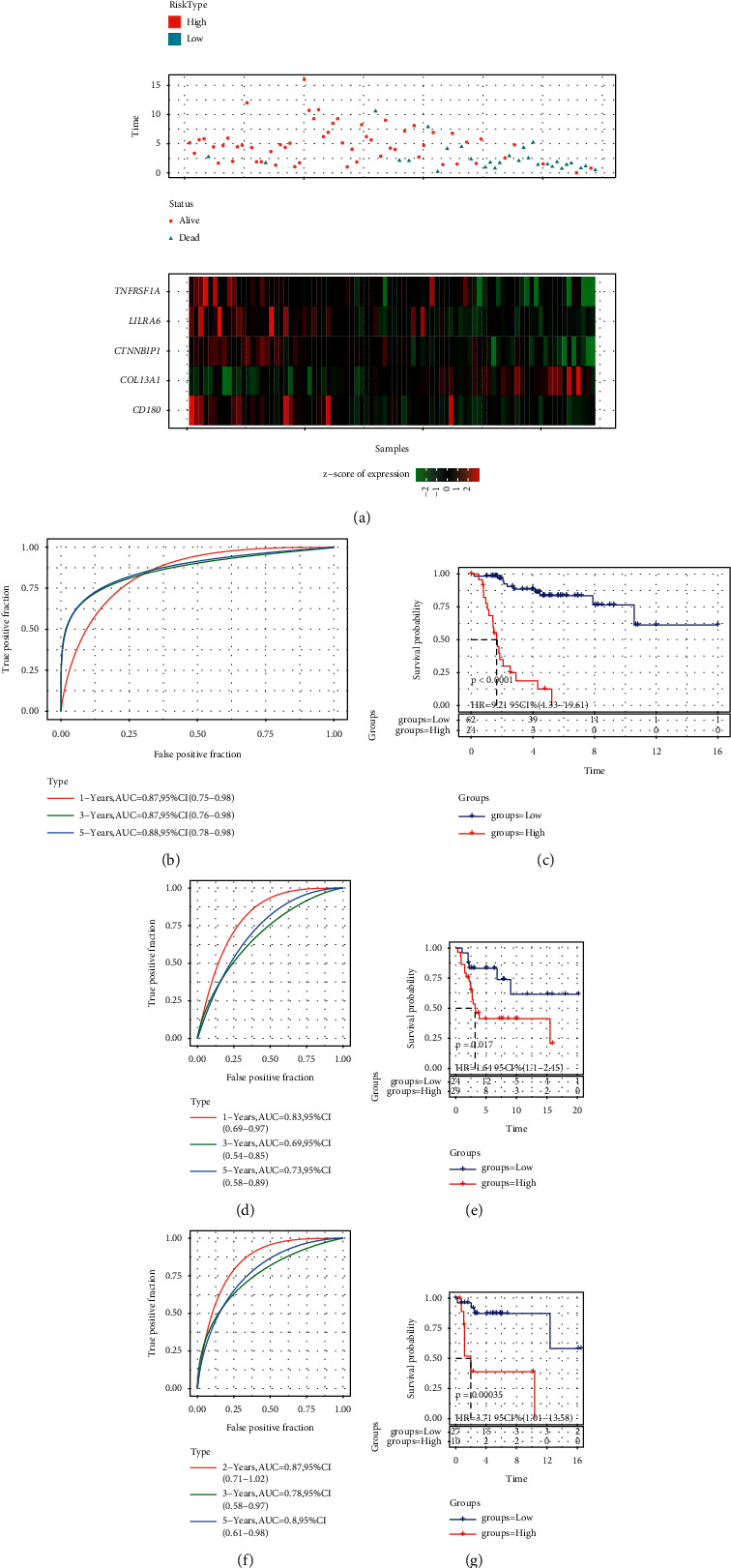
Validating the performance of the 5-gene prognostic model. (a) Risk score, survival status, and z-score expression of prognostic genes of each sample in the TARGET-OS dataset. (b) ROC analysis of the model for predicting 1-year, 3-year, and 5-year overall survival in the TARGET-OS dataset. (c) Kaplan–Meier survival plot of high- and low-PPRS groups in TARGET-OS dataset. (d, e) ROC analysis and survival analysis in GSE21257 dataset. (f, g) ROC analysis and survival analysis in the GSE39055 dataset. The log-rank test was conducted in Kaplan–Meier survival analysis.

For each sample in the TARGET-OS dataset, pyroptosis-related prognostic risk score (PPRS) was calculated. According to the optimal cut-off analyzed by survminer, samples were divided into high-PPRS and low-PPRS groups. We observed that dead samples were significantly enriched in high-PPRS group compared to low-PPRS group (Figure 3(a)). The expression of *COL13A1* was higher in high-PPRS group, while the other four genes were lower expressed (Figure 3(a)). ROC analysis presented that the model was effective to predict 1-year, 3-year, and 5-year overall survival, with a high AUC of 0.87, 0.87, and 0.88 (Figure 3(b)). Kaplan–Meier survival analysis showed that two groups had differential overall survival in the TARGET-OS dataset (*P* < 0.0001, Figure 3(c)). To verify the robustness of the prognostic model, we examined it in another two independent datasets (GSE21257 and GSE39055). Similar results were generated that samples were all classified into two groups with distinct prognoses (*P*=0.017 and *P*=0.00035, respectively, Figures 3(d)–3(g)). The above results demonstrated that the 5-gene prognostic model was valid to predict prognosis for OS patients, and pyroptosis-related genes played an important role in OS development.

### 3.4. The Relation between PPRS Score and Clinical Features

**Figure 4:**
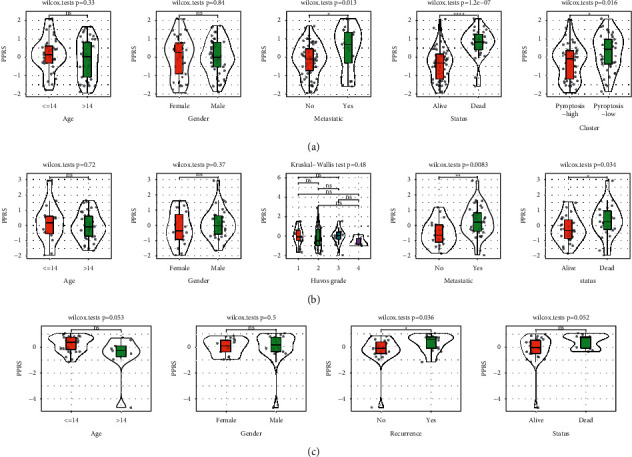
The relation between PPRS score and clinical features in TARGET-OS (a), GSE21257 (b), and GSE39055 (c) datasets. the Wilcoxon test was conducted. ns, no significance. ^*∗*^*P* < 0.05, ^*∗∗*^*P* < 0.01, and ^*∗∗∗∗*^*P* < 0.0001.

We verified that PPRS score was significantly associated with overall survival in both test and validation datasets. To know the relation between PPRS score and other clinical information such as ages, genders, metastasis, and recurrence, we compared their PPRS score between high- and low-PPRS groups. No significant differences in ages, genders, and grades were observed between the two groups in all three datasets (Figures 4(a)–4(c)). Noteworthy, PPRS scores varied significantly between metastatic and nonmetastatic, alive and dead, recurrent and nonrecurrent samples (*P* < 0.05). It could be speculated that pyroptosis-related genes were involved in the cancer cell metastasis.

### 3.5. Differentially Enriched Pathways between High- and Low-PPRS Groups

**Figure 5:**
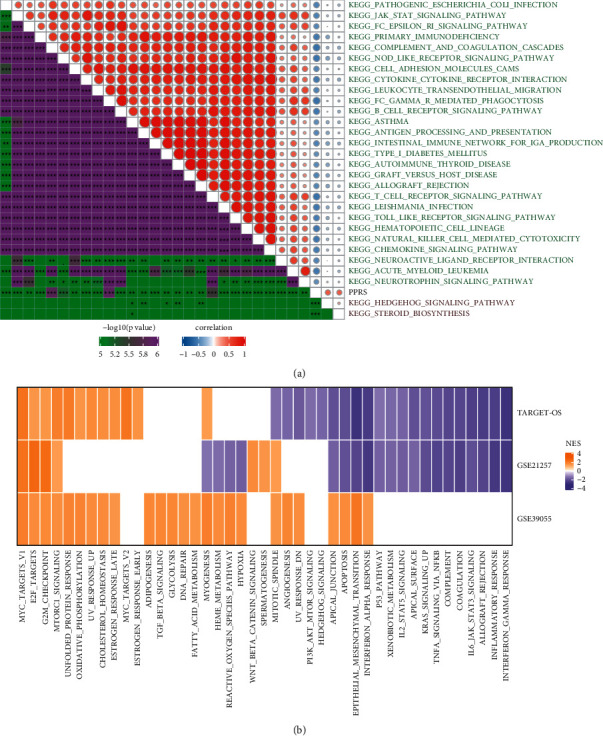
KEGG pathway analysis of high- and low-PPRS groups in the TARGET-OS dataset. (a): Pearson correlation analysis between KEGG pathways and PPRS score. Pathways with |correlation coefficient| ≥ 0.4 were visualized. Blue indicates negative correlation, and red indicates positive correlation. (b) Comparison of enriched pathways between high- and low-PPRS groups in three datasets (FDR <0.05). Yellow indicates higher enrichment in the high-PPRS group, and purple is the reverse. NES, normalized enrichment score. ^*∗*^*P* < 0.05, ^*∗∗*^*P* < 0.01, and ^*∗∗∗*^*P* < 0.001.

To understand the enriched pathways of high- and low-PPRS groups, we calculated ssGSEA score of hallmark pathways for each sample in TARGET-OS dataset. Pearson correlation analysis revealed that 29 pathways were significantly correlated with PPRS score (|R| ≥ 0.4, Figure 5(a)). The majority of pathways were related to immunity such as primary immunodeficiency, complement and coagulation cascades, cytokine-cytokine receptor interaction, B cell receptor signaling pathway, T cell receptor signaling pathway, and chemokine signaling pathway. In the comparison of enriched pathways between high- and low-PPRS groups, cell cycle-related pathways such as Myc targets, E2F targets, and G2M checkpoint were more enriched in the high-PPRS group, while immune-related pathways such as interferon-*γ* response, inflammatory response, and IL6-JAK-STAT3 signaling pathway were more enriched in the low-PPRS group (Figure 5(b)). The above results suggested that the high-PPRS group had higher activity in the cell cycle and may thus contribute to cancer cell invasion and migration. The activation of immune-related pathways in the low-PPRS group possibly served as protective factors for inhibiting cancer cell progression.

### 3.6. TME Features and Immunotherapy/Chemotherapy Response of High- and Low-PPRS Groups

Next, we evaluated whether there was a difference in TME features between the two groups. CIBERSORT analysis revealed a similar distribution of 22 immune cells in two groups (Supplementary Figure S4A). However, ESTIMATE analysis showed that the low-PPRS group had higher stromal and immune infiltration than the high-PPRS group in the TARGET-OS dataset (*P* < 0.01, Supplementary Figure S4B). In GSE21257 and GSE39055 datasets, we observed similar results (Supplementary Figure S5). In addition, we assessed the correlation between the PPRS score and the enrichment of 22 immune cells. CD8 T cells and activated memory CD4 T cells were negatively correlated with PPRS score. M0 macrophages and resting dendritic cells were positively correlated with PPRS score.

Of the immune checkpoints, we found that 6 of 21 were differentially expressed between high- and low-PPRS groups, including CD27, CD47, GEM, HAVCR2, LAG3, and TNFSF4 (*P* < 0.05, Supplementary Figure S6A). Furthermore, we assessed the enrichment of three immunosuppressive cells in two groups. Myeloid-derived suppressor cells (MDSCs) and tumor-associated macrophages (TAMs) were more enriched in the high-PPRS group, while cancer-associated fibroblasts (CAFs) were more enriched in the low-PPRS group (Supplementary Figure S6B). TIDE analysis showed that the low-PPRS group had more serious T cell exclusion and dysfunction than the high-PPRS group. A higher TIDE score was shown in the low-PPRS group, indicating a higher possibility to escape from immune checkpoint blockade therapy, although there was no significance between the two groups (Supplementary Figure S6B). Moreover, in the response to chemotherapy, the PPRS-high group was more sensitive to doxorubicin than the PPRS-low group (Supplementary Figure S7). However, the estimated IC50 of the other three drugs (cisplatin, methotrexate, and paclitaxel) showed no significant difference between the two groups.

### 3.7. Optimizing the Prognostic Model for Clinical Use

**Figure 6:**
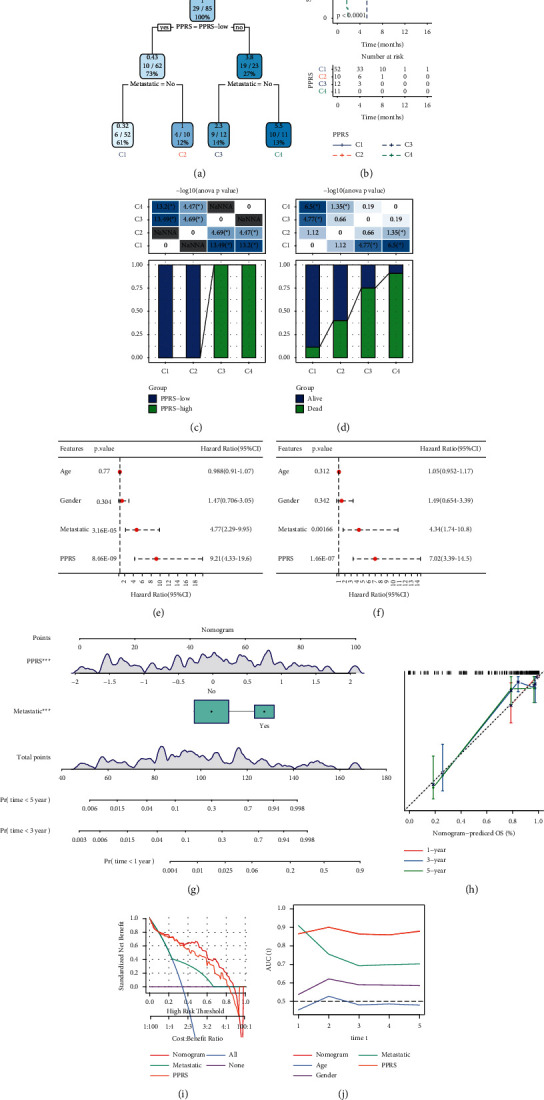
Application of the 5-gene prognostic model in the TARGET-OS dataset. (a) A decision tree based on the prognostic model and metastasis. (b) Kaplan–Meier survival curve of C1 to C4 subgroups. (c, d) The distribution in high- and low-PPRS groups and alive and dead groups in C1 to C4 subgroups. ANOVA was conducted. (e, f) Univariate and multivariate Cox regression analyses on ages, genders, metastasis, and PPRS score. (g) A nomogram based on PPRS score and metastasis. (h) Correction for predicted 1-year, 3-year, and 5-year overall survival based on the observed survival. (i) Decision curve analysis (DCA) of the nomogram, metastasis, and PPRS score. (j): AUC values of the nomogram, ages, genders, metastasis, and PPRS score. The log-rank test was conducted in Kaplan–Meier survival analysis (b) and univariate and multivariate Cox regression analysis (e, f). ANOVA test was conducted in (c, d). ^*∗*^*P* < 0.05.

To make the prognostic model more accurate for clinical use, we constructed a decision tree based on ages, genders, metastasis, and the model. Finally, only metastasis and the model remained, and four subgroups were generated (C1 to C4, Figure 6(a)). Four subgroups varied massively in overall survival, where C1 had the longest survival and C4 had the worst prognosis (Figure 6(b)). Low-PPRS samples were only displayed in C1 and C2 subgroups, and high-PPRS samples were only included in C3 and C4 subgroups (Figures 6(a) and 6(c)). Dead samples were the most distributed in the C4 subgroup and alive samples composed the most in the C1 subgroup, which was consistent with the survival analysis (Figures 6(b) and 6(d)). Univariate and multivariate Cox regression analysis showed that metastasis and PPRS score were independent risk factors (Figures 6(e) and 6(f)). According to PPRS score and metastasis, we established a nomogram to predict 1-year, 3-year, and 5-year prognosis for osteosarcoma patients (Figure 6(g)). The predicted 1-year, 3-year, and 5-year overall survival were corrected (Figure 6(h)). Compared with metastasis and PPRS score, the nomogram was optimal to assist decision-making and create the maximum benefit for patients (Figure 6(i)). ROC analysis showed that the PPRS score and the nomogram had the highest AUC (Figure 6(j)), which further proved the effectiveness and practicability of the nomogram for its application in the clinic.

## 4. Discussion

This study demonstrated that pyroptosis was associated with the overall survival of osteosarcoma, suggesting that pyroptosis played an important role in OS progression. By using the WGCNA methodology, we identified a gene module significantly correlated with pyroptosis. Within the gene module, we screened 187 genes related to pyroptosis and OS prognosis. To construct a prognostic model, LASSO and stepAIC were applied to decrease the number of these genes. Finally, a 5-gene prognostic model consisting of *COL13A1*, *TNFRSF1A*, *LILRA6*, *CTNNBIP1*, and *CD180* was established for predicting the prognosis of osteosarcoma. The 5-gene signature could divide OS samples into high- and low-PPRS groups with distinct overall survival in three independent datasets.

Pyroptosis has been reported to occur with a strong inflammatory response, which is activated by inflammasomes such as NLR family pyrin domain containing 1 (NLRP1) [24], NLRP3 [25], and NOD-like receptor containing a caspase activating and recruitment domain 4 (NLRC4) [26]. By comparing the high-PPRS group to low-PPRS group, we observed differential enrichment of KEGG pathways between them. Noteworthy, a number of immune-related pathways were identified to be negatively associated with PPRS, such as T cell receptor signaling, Toll-like signaling, JAK-STAT signaling, NOD-like receptor signaling, chemokine signaling, and cytokine-cytokine receptor signaling pathways. Our results further demonstrated that the five pyroptosis-related genes were possibly involved in immune-related pathways and the modulation of immunity.

Except for *COL13A1* more expressed in the high-PPRS group, other four prognostic genes were all expressed low in the high-PPRS group. For osteosarcoma, *COL13A1* was a risk factor and the other four genes were protective factors. In research of identifying survival-related genes in osteosarcoma, *COL13A1* and *CTNNBIP1* were also included as prognostic biomarkers [27]. *COL13A1* was upregulated and *CTNNBIP1* was downregulated in dead OS patients, which was consistent with our result that *COL13A1* overexpression and *CTNNBIP1* downregulation were associated with unfavorable prognosis.

In bladder cancer, Miyake et al. found that a high uterine level of *COL13A1* was associated with a poor prognosis [28]. They discovered that *COL4A1*+*COL13A1* was an independent predictor for intravesical recurrence of bladder cancer [28]. High expression of *COL13A1* was observed in breast cancer cells, correlated with invasive tumor growth, and induced anoikis resistance [29]. *CTNNBIP1* was reported as a suppressor in lung cancer that high expression of *CTNNBIP1* could inhibit the progression of lung cancer [30]. Low expression of *CTNNBIP1* was a risk factor for lung cancer (hazard ratio = 1.85) [30], which is accordant with our observation that the low-PPRS group had lower expression of *CTNNBIP1*. *CTNNBIP1* downregulation was also discovered in human gastric adenocarcinoma tissues [31].

*TNFRSF1A* encodes a transmembrane receptor for tumor necrosis factor (TNF)-*α*. High expression of *TNFRSF1A* was demonstrated to be associated with STAT3 activation in breast cancer cells, where STAT3 is known as a critical factor in tumorigenesis [32]. *CD180* was identified as a pharmacodynamic biomarker for tumors especially in lymphocytic leukemia [33]. *LILRA6* has not been reported to be significantly associated with cancer development, serving as a new potential biomarker for predicting OS prognosis.

Besides differentially enriched pathways, the high-PPRS and low-PPRS groups also had a difference in immune infiltration. Higher immune infiltration was shown in the low-PPRS group due to a more activated immune response in the low-PPRS group. In addition, we constructed a decision tree based on PPRS and metastasis. Four subgroups (C1–C4) were classified by the decision tree with differential prognoses. For the application of the 5-gene signature, we established a nomogram presenting superior performance than PPRS only for predicting OS prognosis.

In conclusion, this study identified five prognostic genes related to pyroptosis and constructed a 5-gene signature with robust performance in three independent datasets. We further demonstrated the important role of pyroptosis in osteosarcoma development and the relation between pyroptosis and immunity. The five prognostic pyroptosis-associated genes may play an important role in osteosarcoma pyroptosis. The 5-gene signature could serve as a new tool for predicting OS prognosis. In addition, the five prognostic genes may be potential targets for exploring new molecular therapies for OS patients.

## Data Availability

The datasets generated and/or analyzed during the current study are available in the GSE21257 repository (https://www.ncbi.nlm.nih.gov/geo/query/acc.cgi?acc=GSE), in GSE39055 repository (https://www.ncbi.nlm.nih.gov/geo/query/acc.cgi?acc=), and in TARGET repository (https://portal.gdc.cancer.gov/projects/TARGET-OS).

## Supplementary Materials

Supplementary Materials
*Supplementary Figure S1*. KEGG and GO function analysis on genes within the purple module. (A) The top 10 enriched KEGG pathways. (B–D) The top 10 enriched GO terms of molecular function, cellular component, and biological process. *Supplementary Figure S2*. Identifying prognostic genes related to pyroptosis and constructing a prognostic model. (A) Identifying genes in the purple module was significantly associated with prognosis by univariate Cox regression analysis. Log-rank test was conducted. (B–C) LASSO Cox regression analysis for decreasing the number of genes. The dotted red line indicates the optimal lambda value of 0.1395. (D) The LASSO coefficients of five prognostic genes. *Supplementary Figure S3*. Comparison of TME between high- and low-PPRS groups in TARGET-OS dataset. (A) The proportion of 22 immune cells in two groups. Student's *t*-test was conducted. (B) Comparison of the stromal score, immune score, and ESTIMATE score between high- and low-PPRS groups. Student's *t*-test was conducted. (C) Pearson correlation analysis between PPRS score and enrichment of immune cells. Blue and red indicate negative and positive correlations, respectively. ns, not significant. ^*∗*^*P* < 0.05, ^*∗∗*^*P* < 0.01, and ^*∗∗∗*^*P* < 0.001. *Supplementary Figure S4.* Comparison of TME in GSE21257 (A-B) and GSE39055 (C-D) datasets. ns, not significant. ^*∗*^*P* < 0.05, ^*∗∗*^*P* < 0.01, and ^*∗∗∗*^*P* < 0.001. Supplementary Figure S5. (A) Expression of immune checkpoints in high- and low-PPRS groups. (B) Enrichment of immunosuppressive cells (MDSC, CAF, and M2 TAM), T cell exclusion, T cell dysfunction, and TIDE score in high- and low-PPRS groups. *Supplementary Figure S6.* (A) Expression of immune checkpoints in high- and low-PPRS groups. (B) Enrichment of immunosuppressive cells (MDSC, CAF, and M2 TAM), T cell exclusion, T cell dysfunction, and TIDE score in high- and low-PPRS groups. *Supplementary Figure S7.* The estimated IC50 of four chemotherapeutic drugs in TARGET-OS (A), GSE21257 (B), and GSE39055 (C) datasets.
