# Supplementary figures and images for: Developing a 5-Gene Signature Related to Pyroptosis for Osteosarcoma Patients
Source: J Oncol. 2022 Aug 5;2022:1317990. doi: 10.1155/2022/1317990 (PMC9410960; doi:10.1155/2022/1317990)

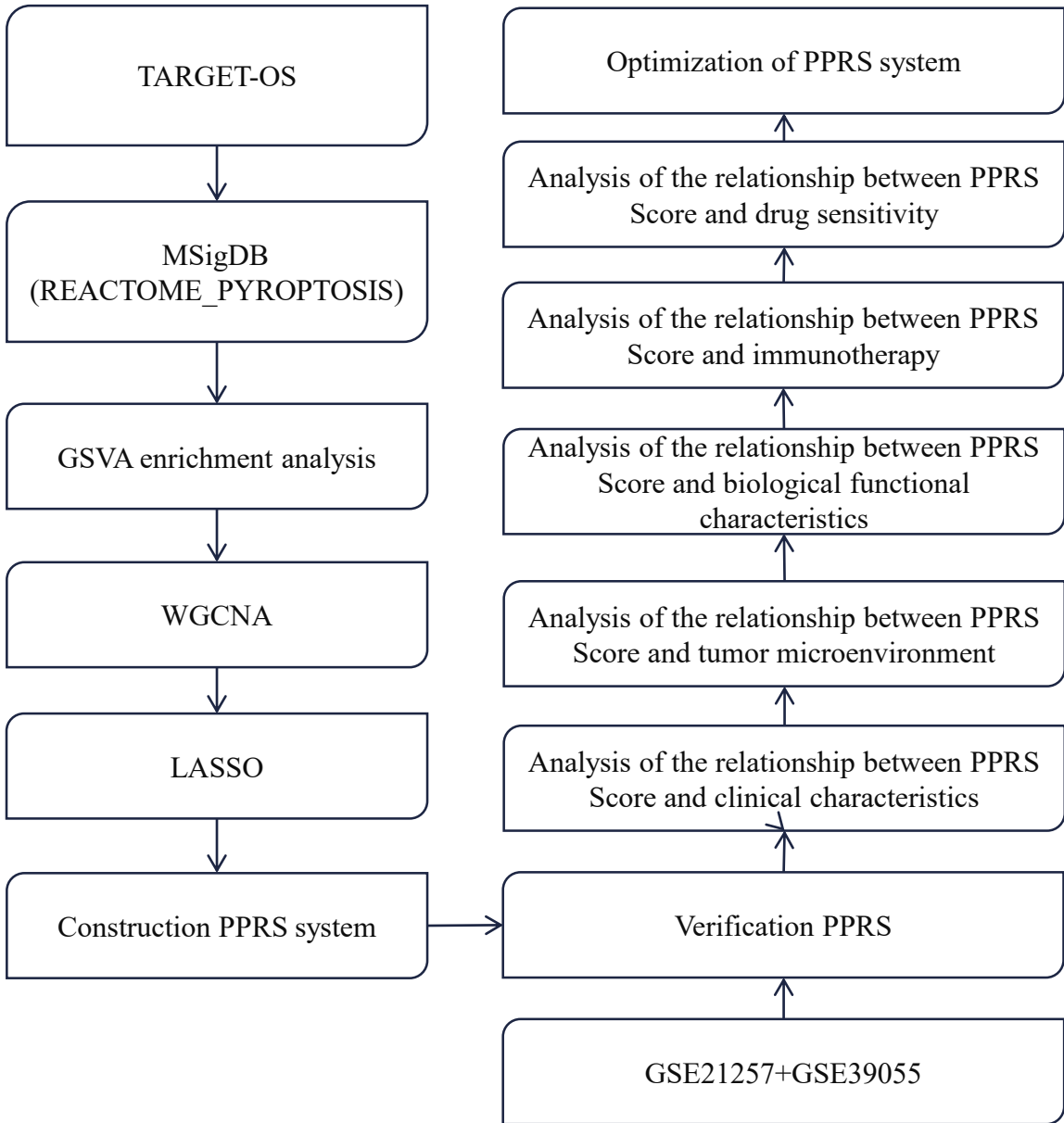

Supplement: Supplementary Materials — Supplementary Figure S1. KEGG and GO function analysis on genes within the purple module. (A) The top 10 enriched KEGG pathways. (B–D) The top 10 enriched GO terms of molecular function, cellular component, and biological process. Supplementary Figure S2. Identifying prognostic genes related to pyroptosis and constructing a prognostic model. (A) Identifying genes in the purple module was significantly associated with prognosis by univariate Cox regression analysis. Log-rank test was conducted. (B–C) LASSO Cox regression analysis for decreasing the number of genes. The dotted red line indicates the optimal lambda value of 0.1395. (D) The LASSO coefficients of five prognostic genes. Supplementary Figure S3. Comparison of TME between high- and low-PPRS groups in TARGET-OS dataset. (A) The proportion of 22 immune cells in two groups. Student's t-test was conducted. (B) Comparison of the stromal score, immune score, and ESTIMATE score between high- and low-PPRS groups. Student's t-test was conducted. (C) Pearson correlation analysis between PPRS score and enrichment of immune cells. Blue and red indicate negative and positive correlations, respectively. ns, not significant. ∗P < 0.05, ∗∗P < 0.01, and ∗∗∗P < 0.001. Supplementary Figure S4. Comparison of TME in GSE21257 (A-B) and GSE39055 (C-D) datasets. ns, not significant. ∗P < 0.05, ∗∗P < 0.01, and ∗∗∗P < 0.001. Supplementary Figure S5. (A) Expression of immune checkpoints in high- and low-PPRS groups. (B) Enrichment of immunosuppressive cells (MDSC, CAF, and M2 TAM), T cell exclusion, T cell dysfunction, and TIDE score in high- and low-PPRS groups. Supplementary Figure S6. (A) Expression of immune checkpoints in high- and low-PPRS groups. (B) Enrichment of immunosuppressive cells (MDSC, CAF, and M2 TAM), T cell exclusion, T cell dysfunction, and TIDE score in high- and low-PPRS groups. Supplementary Figure S7. The estimated IC50 of four chemotherapeutic drugs in TARGET-OS (A), GSE21257 (B), and GSE39055 (C) datasets. [file 1317990.f1.zip › Supplementary Figure S1 (1).pdf]

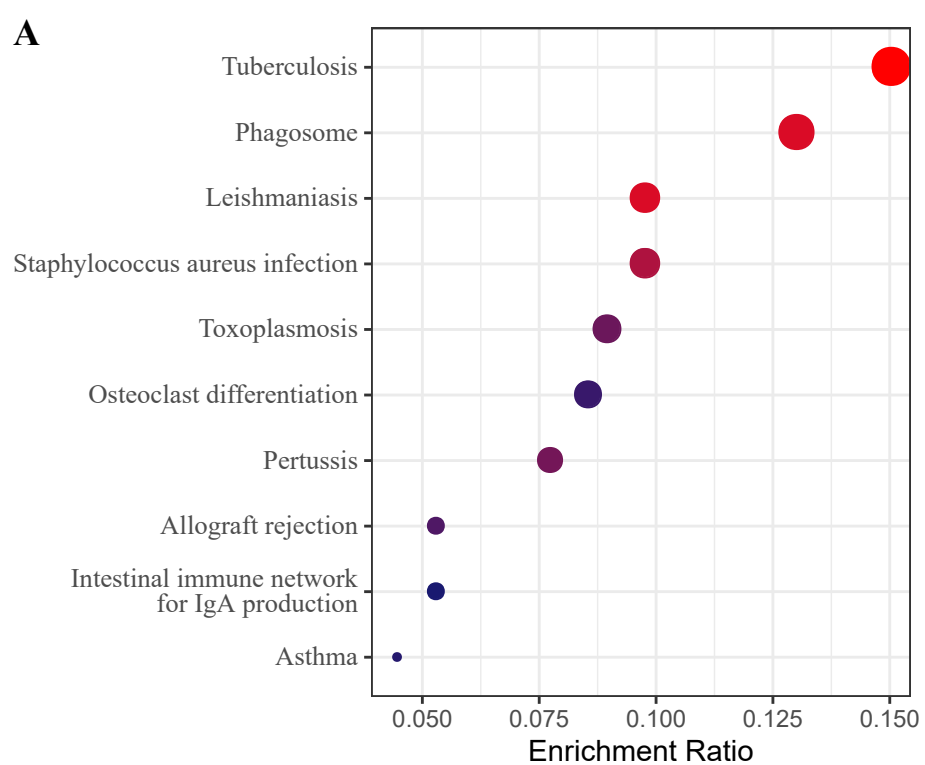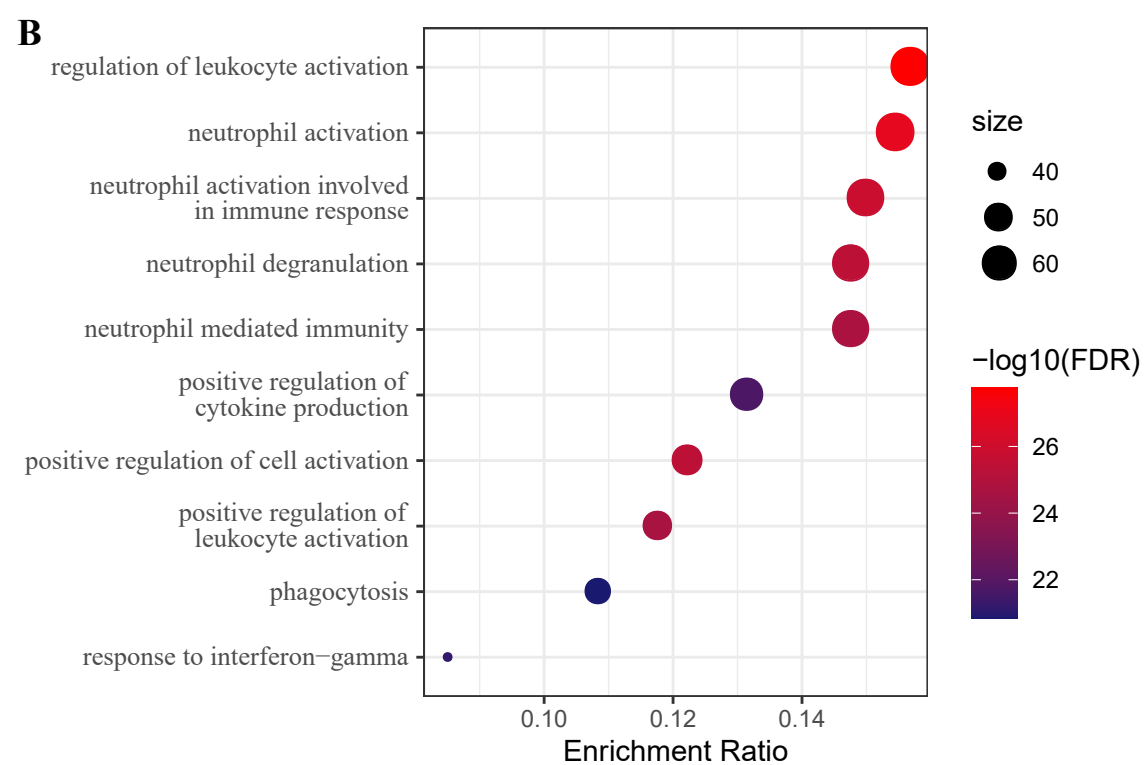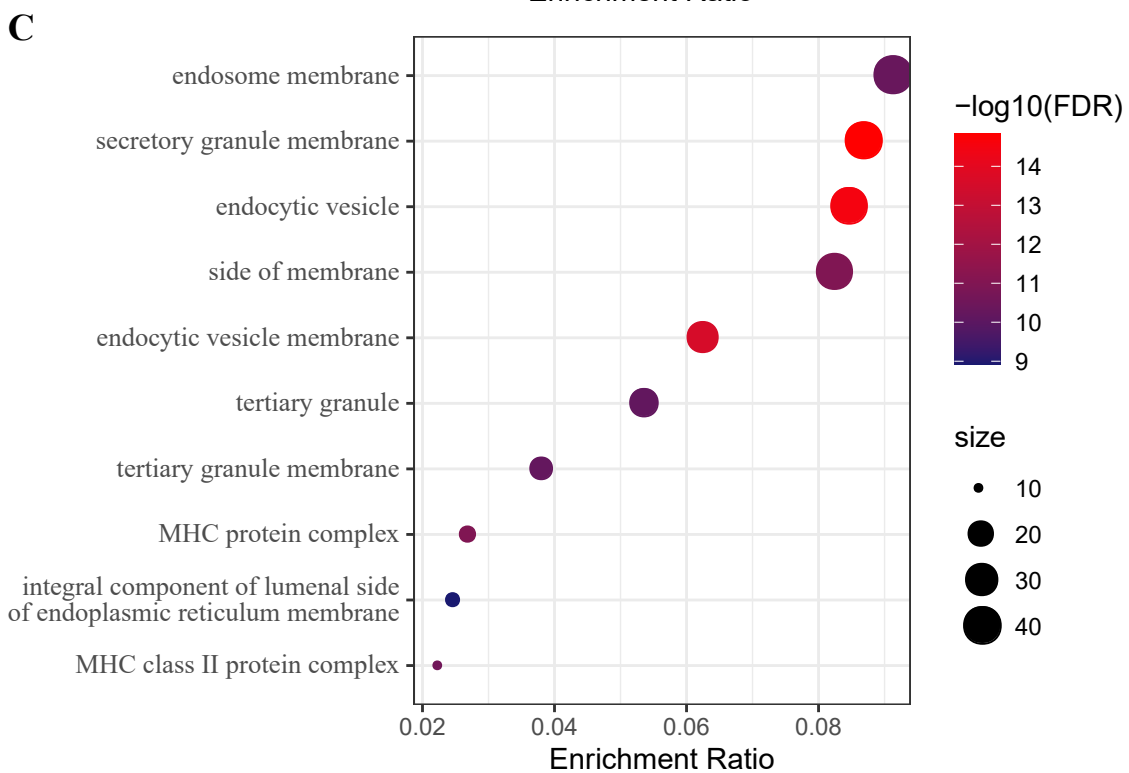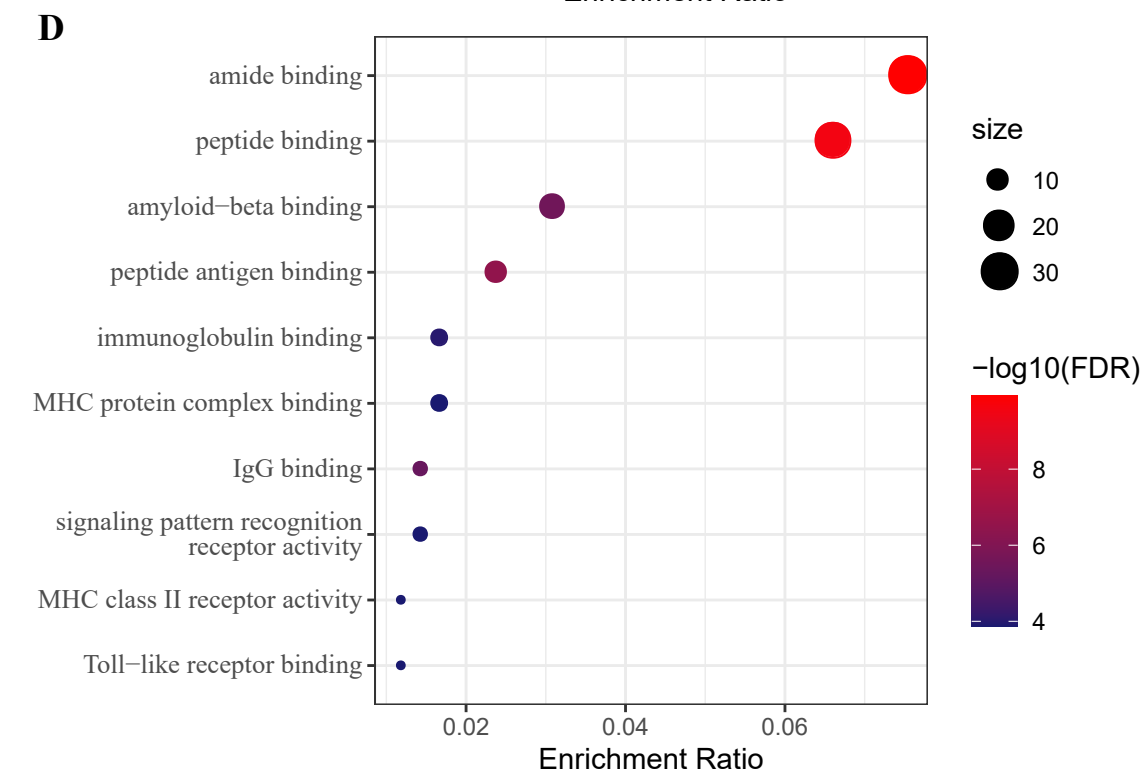

Supplement: Supplementary Materials — Supplementary Figure S1. KEGG and GO function analysis on genes within the purple module. (A) The top 10 enriched KEGG pathways. (B–D) The top 10 enriched GO terms of molecular function, cellular component, and biological process. Supplementary Figure S2. Identifying prognostic genes related to pyroptosis and constructing a prognostic model. (A) Identifying genes in the purple module was significantly associated with prognosis by univariate Cox regression analysis. Log-rank test was conducted. (B–C) LASSO Cox regression analysis for decreasing the number of genes. The dotted red line indicates the optimal lambda value of 0.1395. (D) The LASSO coefficients of five prognostic genes. Supplementary Figure S3. Comparison of TME between high- and low-PPRS groups in TARGET-OS dataset. (A) The proportion of 22 immune cells in two groups. Student's t-test was conducted. (B) Comparison of the stromal score, immune score, and ESTIMATE score between high- and low-PPRS groups. Student's t-test was conducted. (C) Pearson correlation analysis between PPRS score and enrichment of immune cells. Blue and red indicate negative and positive correlations, respectively. ns, not significant. ∗P < 0.05, ∗∗P < 0.01, and ∗∗∗P < 0.001. Supplementary Figure S4. Comparison of TME in GSE21257 (A-B) and GSE39055 (C-D) datasets. ns, not significant. ∗P < 0.05, ∗∗P < 0.01, and ∗∗∗P < 0.001. Supplementary Figure S5. (A) Expression of immune checkpoints in high- and low-PPRS groups. (B) Enrichment of immunosuppressive cells (MDSC, CAF, and M2 TAM), T cell exclusion, T cell dysfunction, and TIDE score in high- and low-PPRS groups. Supplementary Figure S6. (A) Expression of immune checkpoints in high- and low-PPRS groups. (B) Enrichment of immunosuppressive cells (MDSC, CAF, and M2 TAM), T cell exclusion, T cell dysfunction, and TIDE score in high- and low-PPRS groups. Supplementary Figure S7. The estimated IC50 of four chemotherapeutic drugs in TARGET-OS (A), GSE21257 (B), and GSE39055 (C) datasets. [file 1317990.f1.zip › Supplementary Figure S2 (1).pdf]

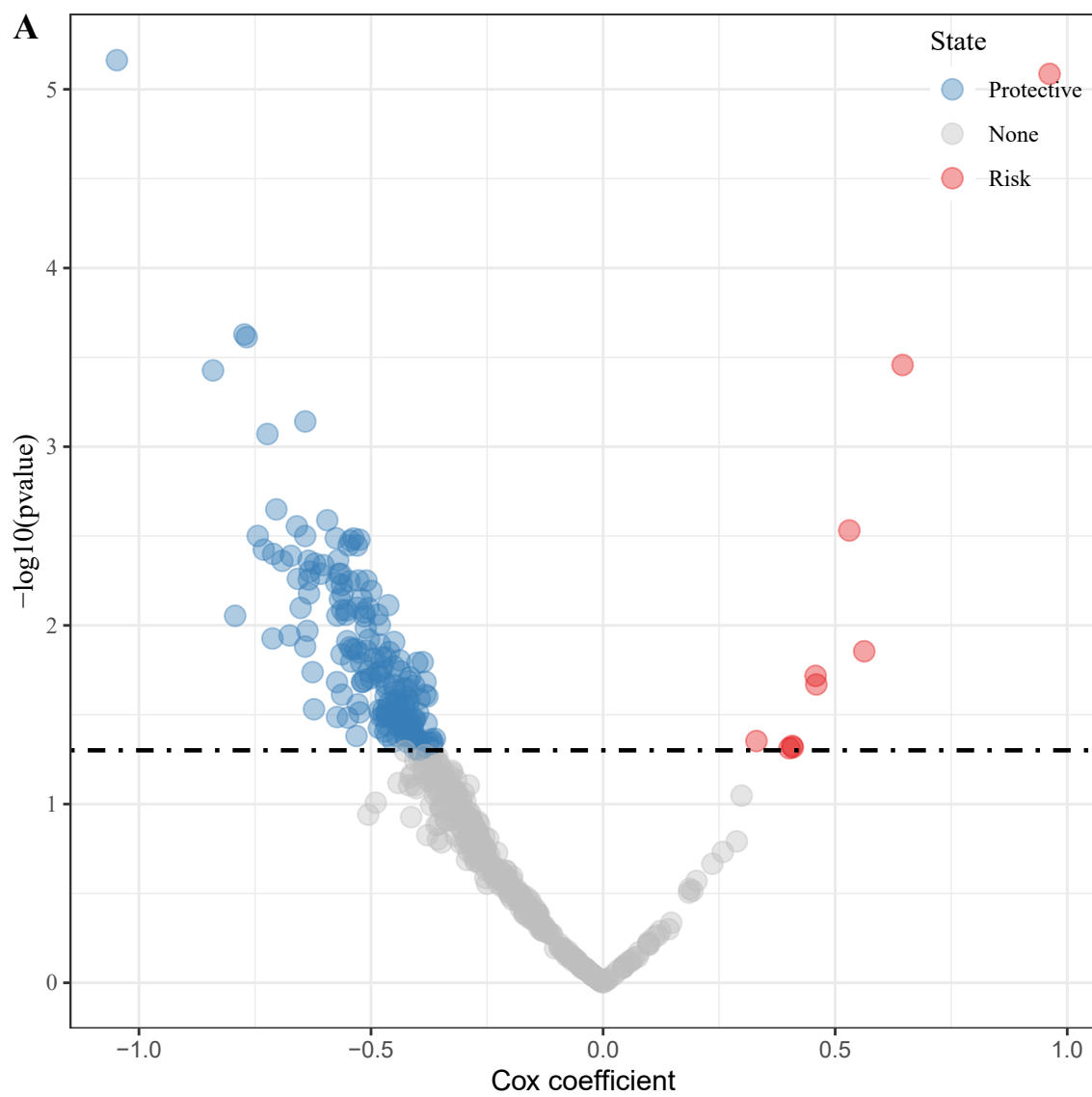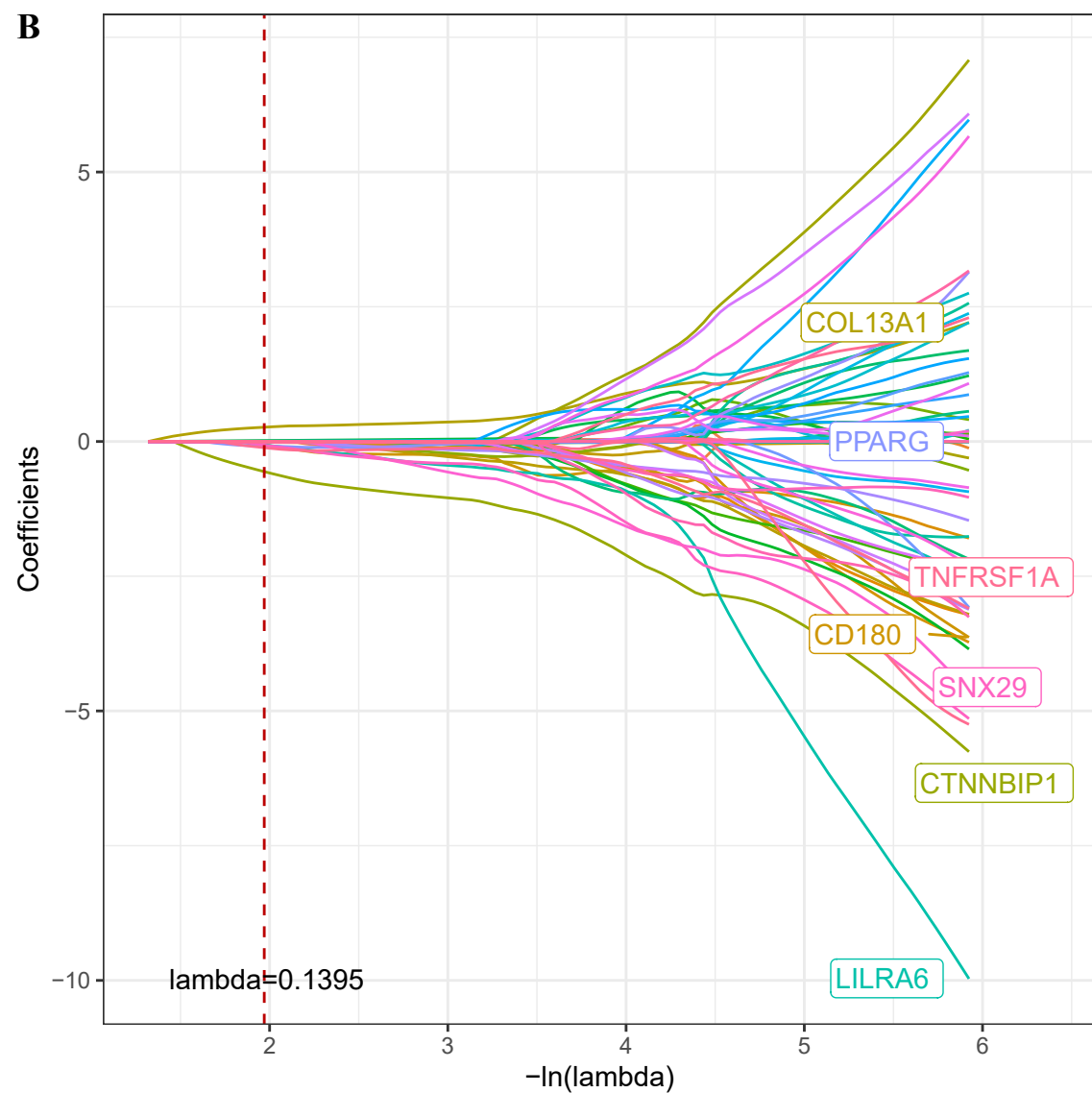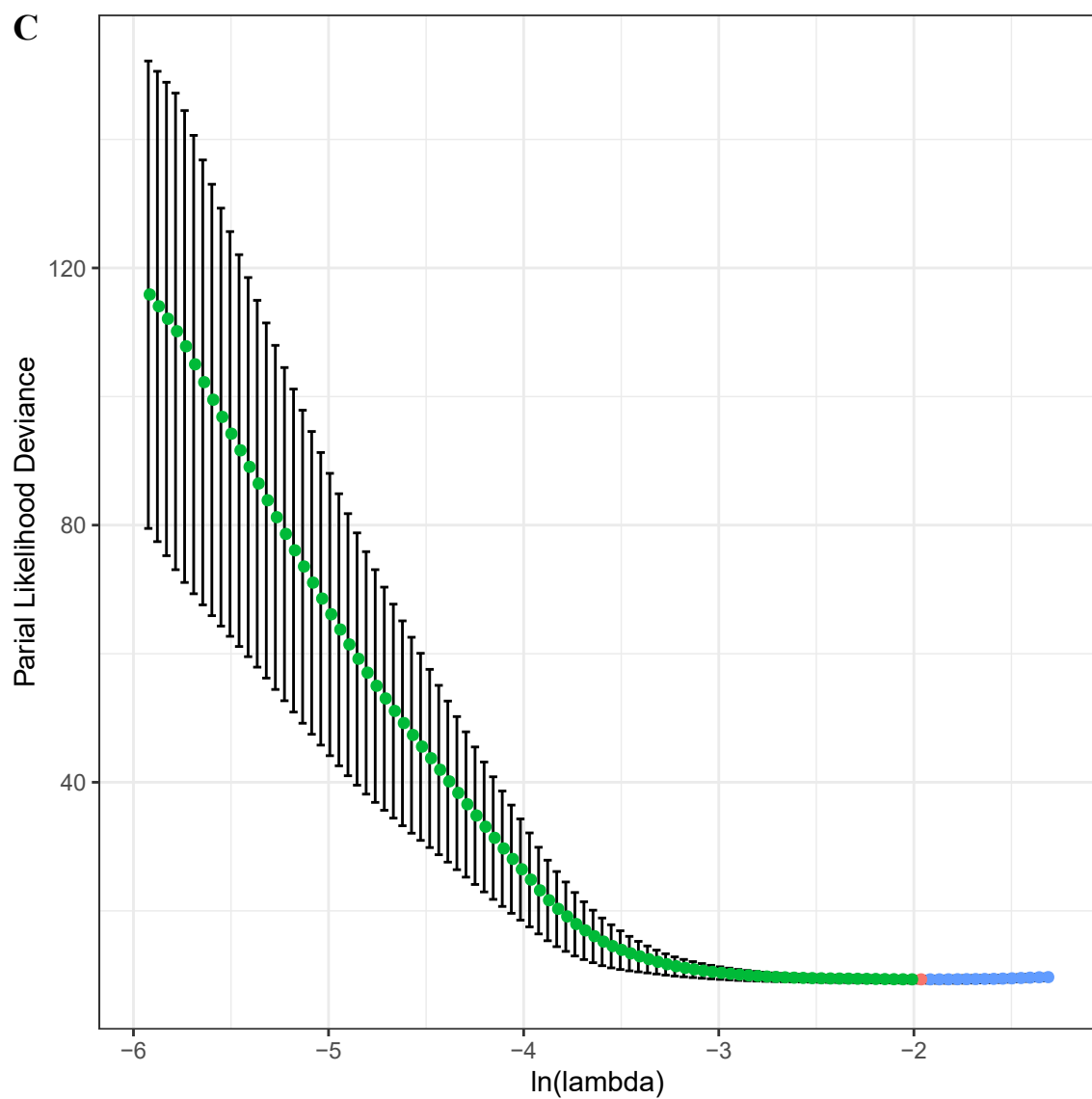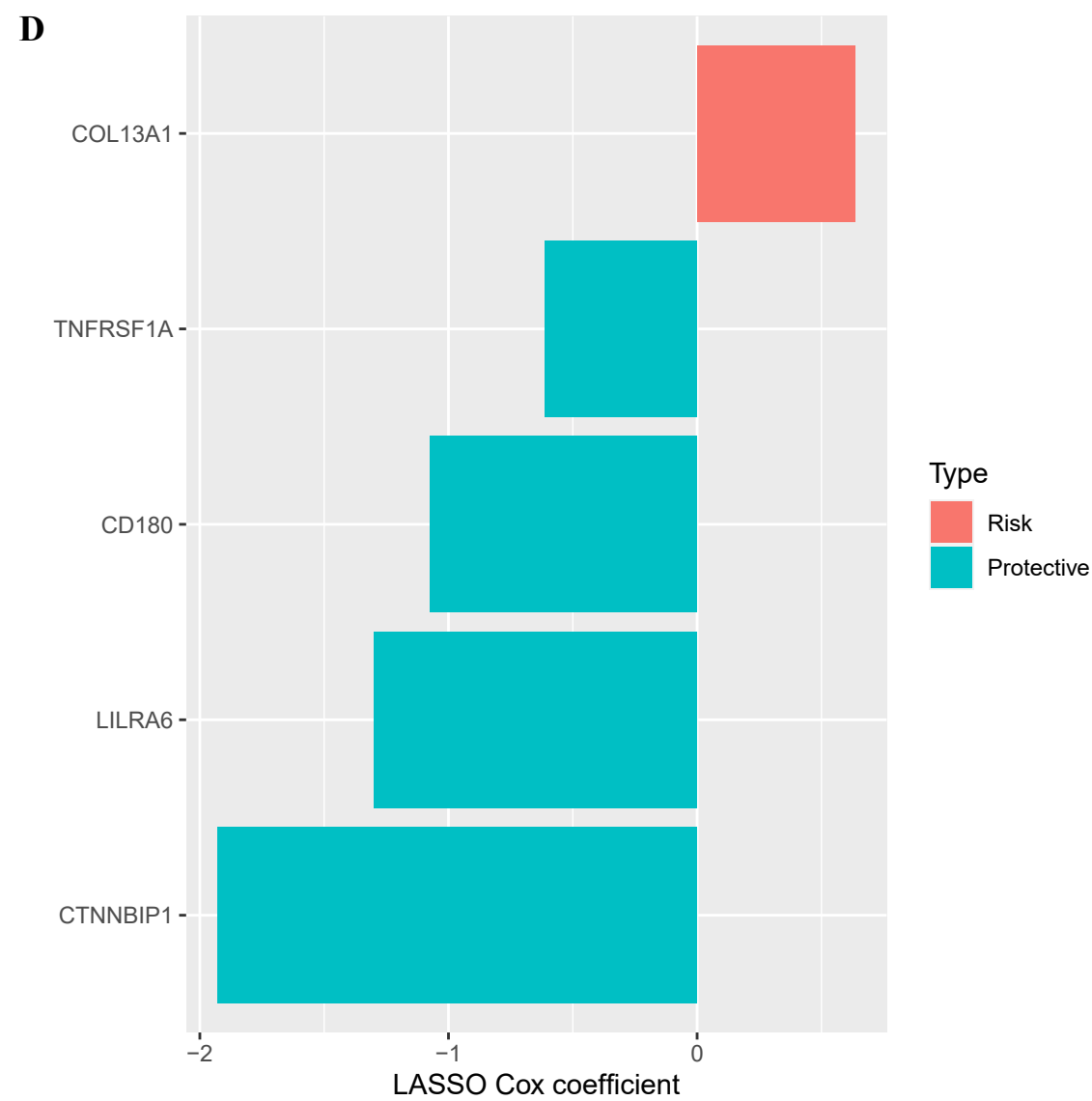

Supplement: Supplementary Materials — Supplementary Figure S1. KEGG and GO function analysis on genes within the purple module. (A) The top 10 enriched KEGG pathways. (B–D) The top 10 enriched GO terms of molecular function, cellular component, and biological process. Supplementary Figure S2. Identifying prognostic genes related to pyroptosis and constructing a prognostic model. (A) Identifying genes in the purple module was significantly associated with prognosis by univariate Cox regression analysis. Log-rank test was conducted. (B–C) LASSO Cox regression analysis for decreasing the number of genes. The dotted red line indicates the optimal lambda value of 0.1395. (D) The LASSO coefficients of five prognostic genes. Supplementary Figure S3. Comparison of TME between high- and low-PPRS groups in TARGET-OS dataset. (A) The proportion of 22 immune cells in two groups. Student's t-test was conducted. (B) Comparison of the stromal score, immune score, and ESTIMATE score between high- and low-PPRS groups. Student's t-test was conducted. (C) Pearson correlation analysis between PPRS score and enrichment of immune cells. Blue and red indicate negative and positive correlations, respectively. ns, not significant. ∗P < 0.05, ∗∗P < 0.01, and ∗∗∗P < 0.001. Supplementary Figure S4. Comparison of TME in GSE21257 (A-B) and GSE39055 (C-D) datasets. ns, not significant. ∗P < 0.05, ∗∗P < 0.01, and ∗∗∗P < 0.001. Supplementary Figure S5. (A) Expression of immune checkpoints in high- and low-PPRS groups. (B) Enrichment of immunosuppressive cells (MDSC, CAF, and M2 TAM), T cell exclusion, T cell dysfunction, and TIDE score in high- and low-PPRS groups. Supplementary Figure S6. (A) Expression of immune checkpoints in high- and low-PPRS groups. (B) Enrichment of immunosuppressive cells (MDSC, CAF, and M2 TAM), T cell exclusion, T cell dysfunction, and TIDE score in high- and low-PPRS groups. Supplementary Figure S7. The estimated IC50 of four chemotherapeutic drugs in TARGET-OS (A), GSE21257 (B), and GSE39055 (C) datasets. [file 1317990.f1.zip › Supplementary Figure S3 (1).pdf]

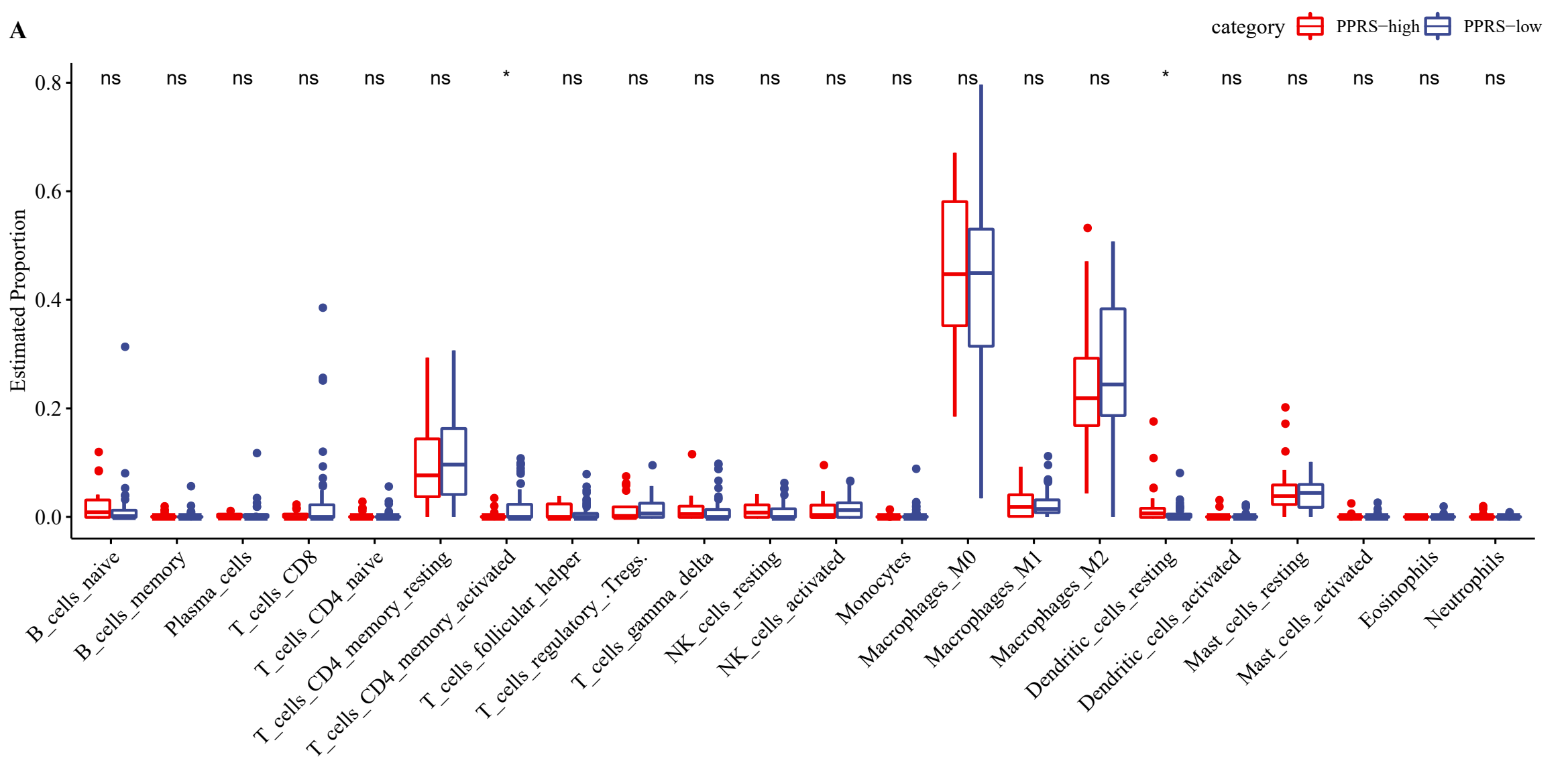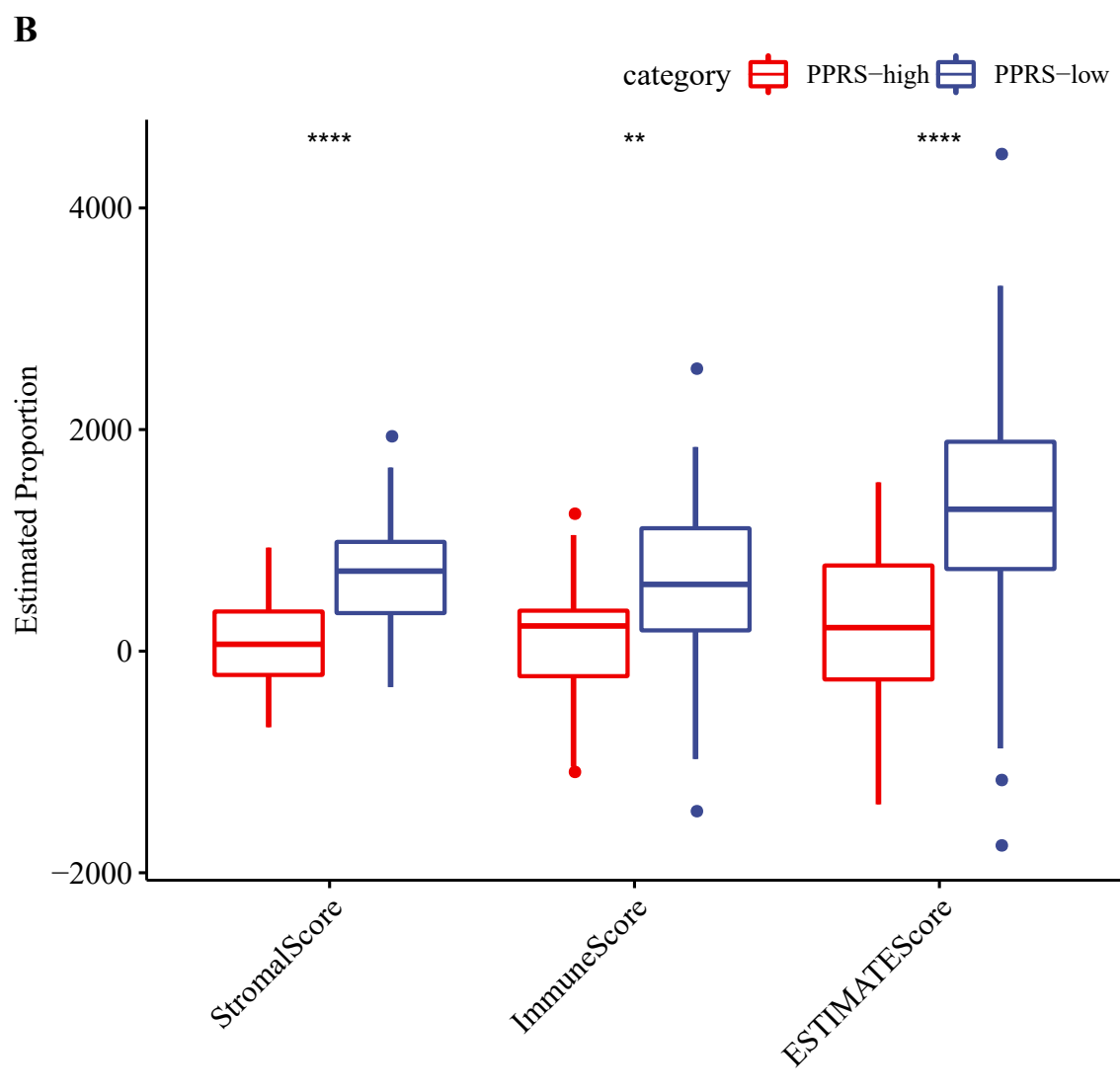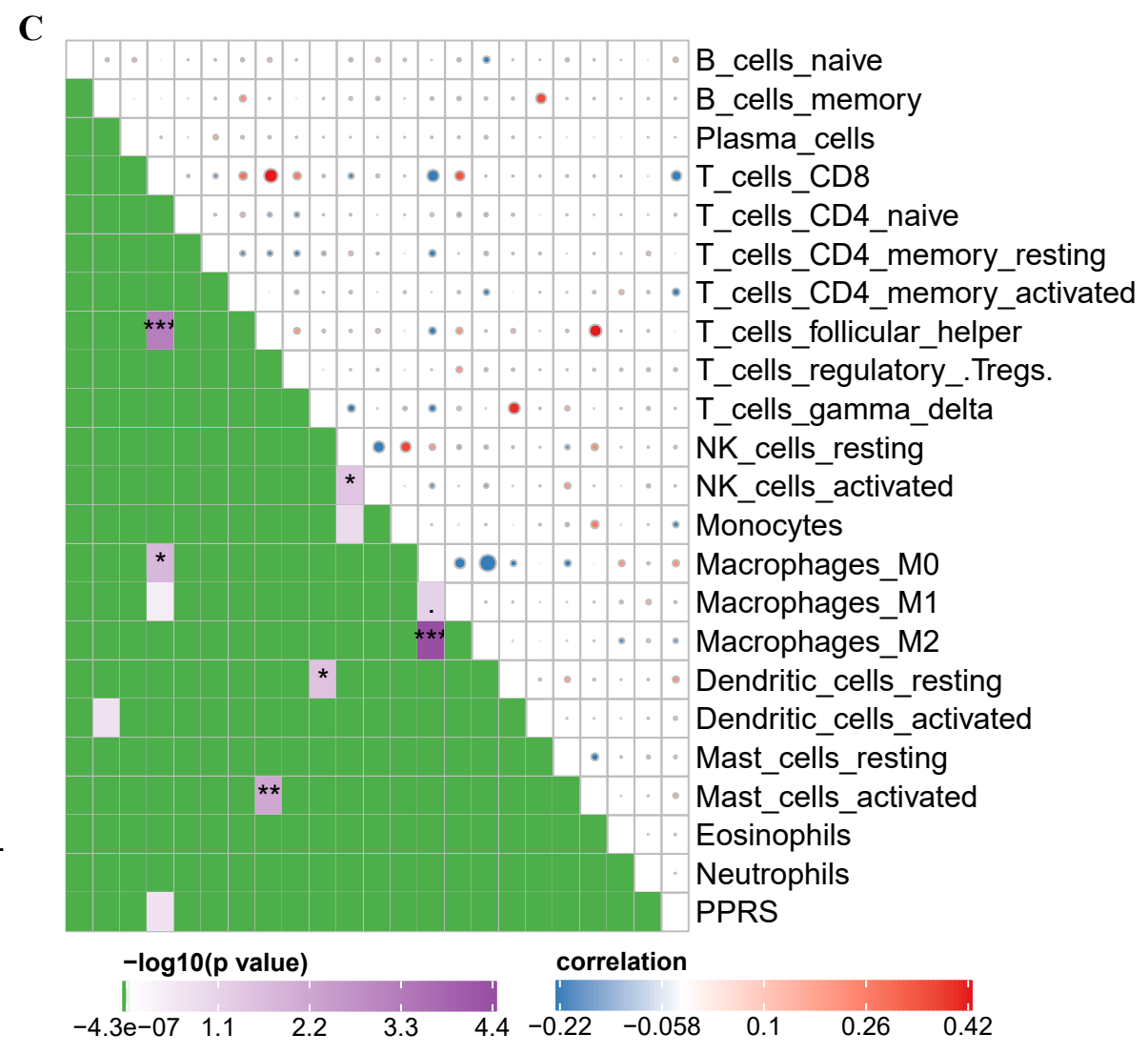

Supplement: Supplementary Materials — Supplementary Figure S1. KEGG and GO function analysis on genes within the purple module. (A) The top 10 enriched KEGG pathways. (B–D) The top 10 enriched GO terms of molecular function, cellular component, and biological process. Supplementary Figure S2. Identifying prognostic genes related to pyroptosis and constructing a prognostic model. (A) Identifying genes in the purple module was significantly associated with prognosis by univariate Cox regression analysis. Log-rank test was conducted. (B–C) LASSO Cox regression analysis for decreasing the number of genes. The dotted red line indicates the optimal lambda value of 0.1395. (D) The LASSO coefficients of five prognostic genes. Supplementary Figure S3. Comparison of TME between high- and low-PPRS groups in TARGET-OS dataset. (A) The proportion of 22 immune cells in two groups. Student's t-test was conducted. (B) Comparison of the stromal score, immune score, and ESTIMATE score between high- and low-PPRS groups. Student's t-test was conducted. (C) Pearson correlation analysis between PPRS score and enrichment of immune cells. Blue and red indicate negative and positive correlations, respectively. ns, not significant. ∗P < 0.05, ∗∗P < 0.01, and ∗∗∗P < 0.001. Supplementary Figure S4. Comparison of TME in GSE21257 (A-B) and GSE39055 (C-D) datasets. ns, not significant. ∗P < 0.05, ∗∗P < 0.01, and ∗∗∗P < 0.001. Supplementary Figure S5. (A) Expression of immune checkpoints in high- and low-PPRS groups. (B) Enrichment of immunosuppressive cells (MDSC, CAF, and M2 TAM), T cell exclusion, T cell dysfunction, and TIDE score in high- and low-PPRS groups. Supplementary Figure S6. (A) Expression of immune checkpoints in high- and low-PPRS groups. (B) Enrichment of immunosuppressive cells (MDSC, CAF, and M2 TAM), T cell exclusion, T cell dysfunction, and TIDE score in high- and low-PPRS groups. Supplementary Figure S7. The estimated IC50 of four chemotherapeutic drugs in TARGET-OS (A), GSE21257 (B), and GSE39055 (C) datasets. [file 1317990.f1.zip › Supplementary Figure S4 (1).pdf]

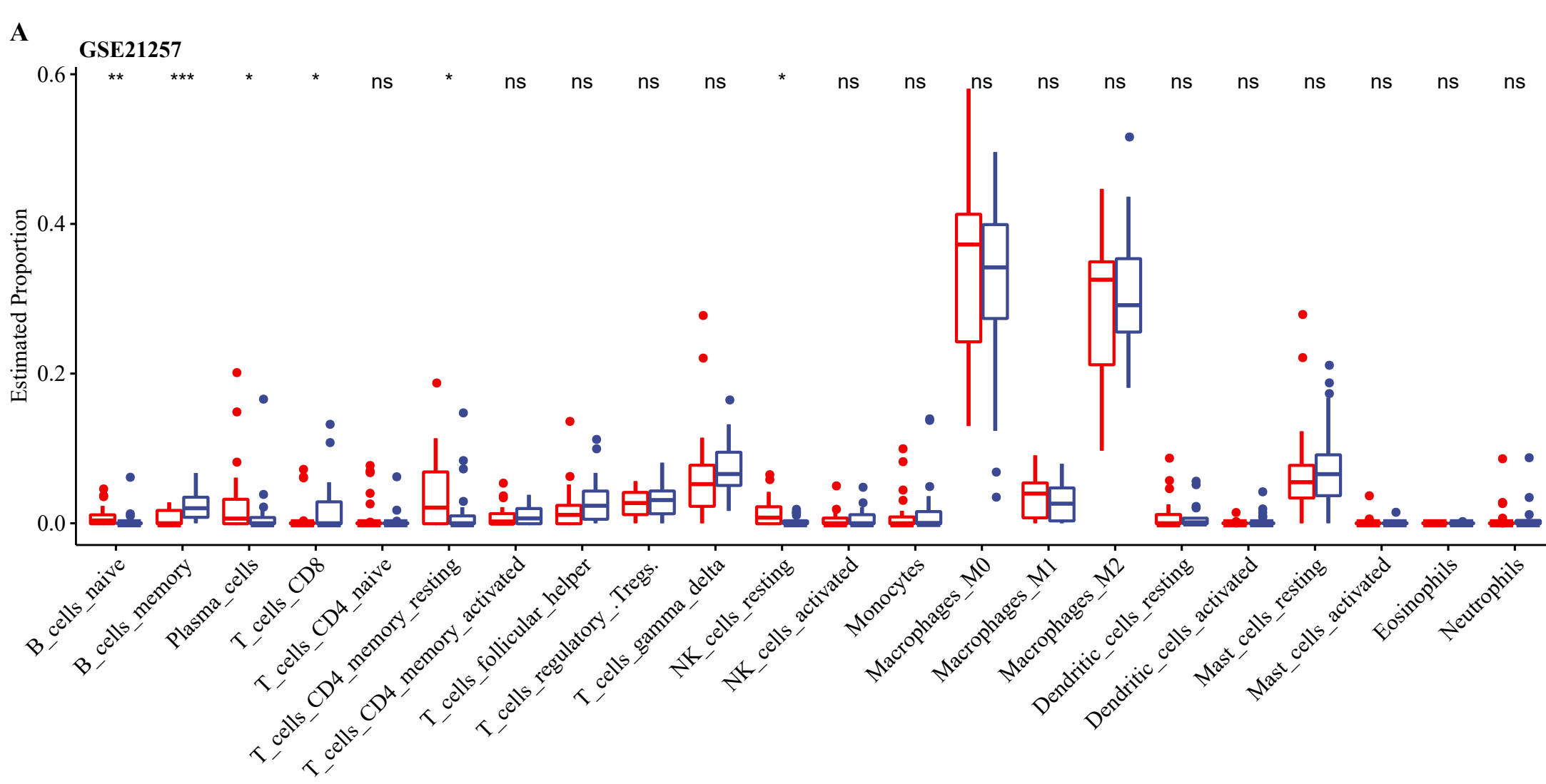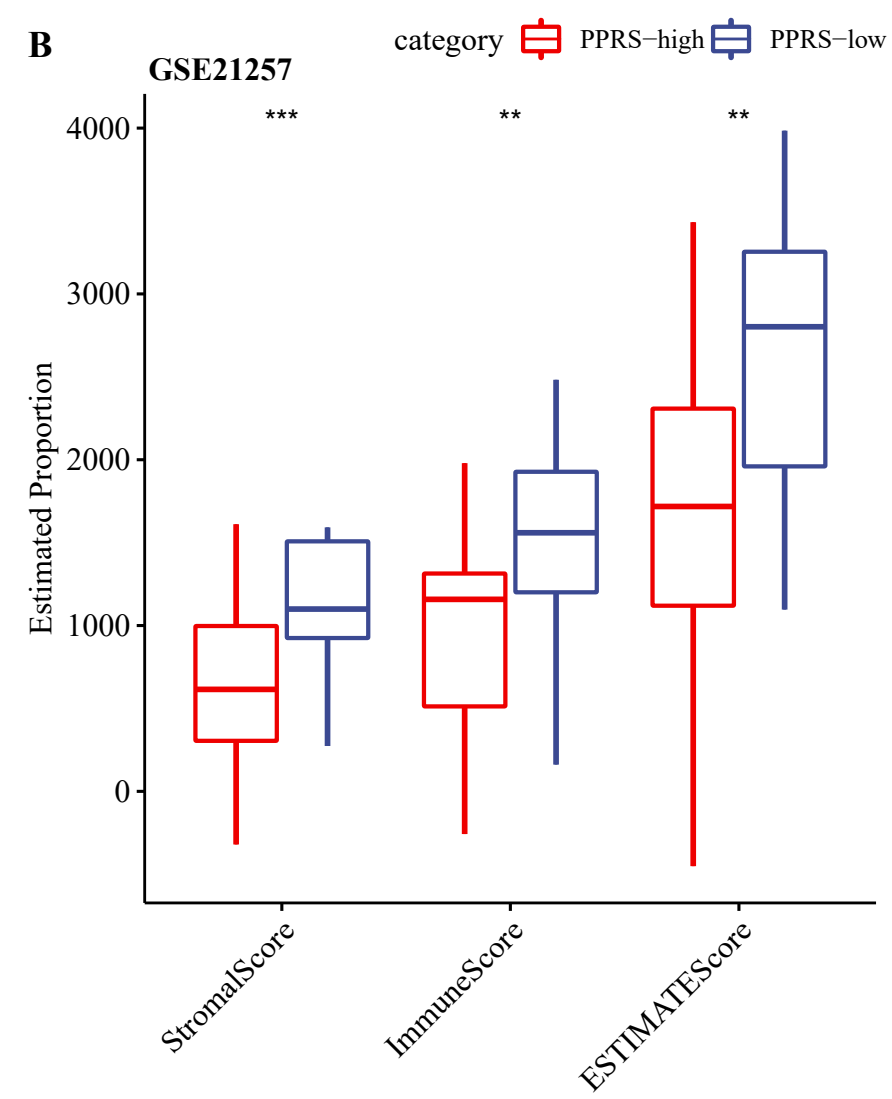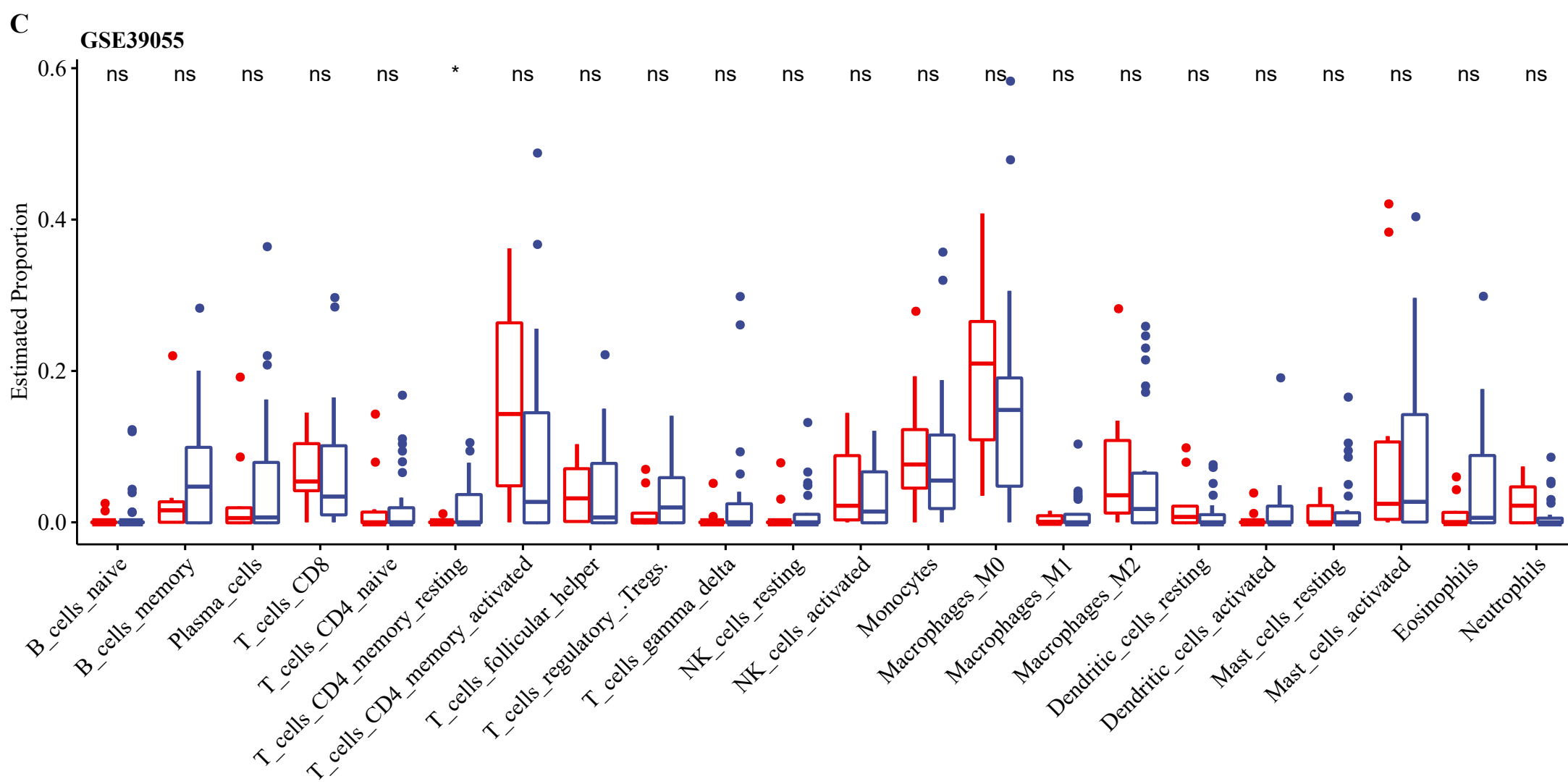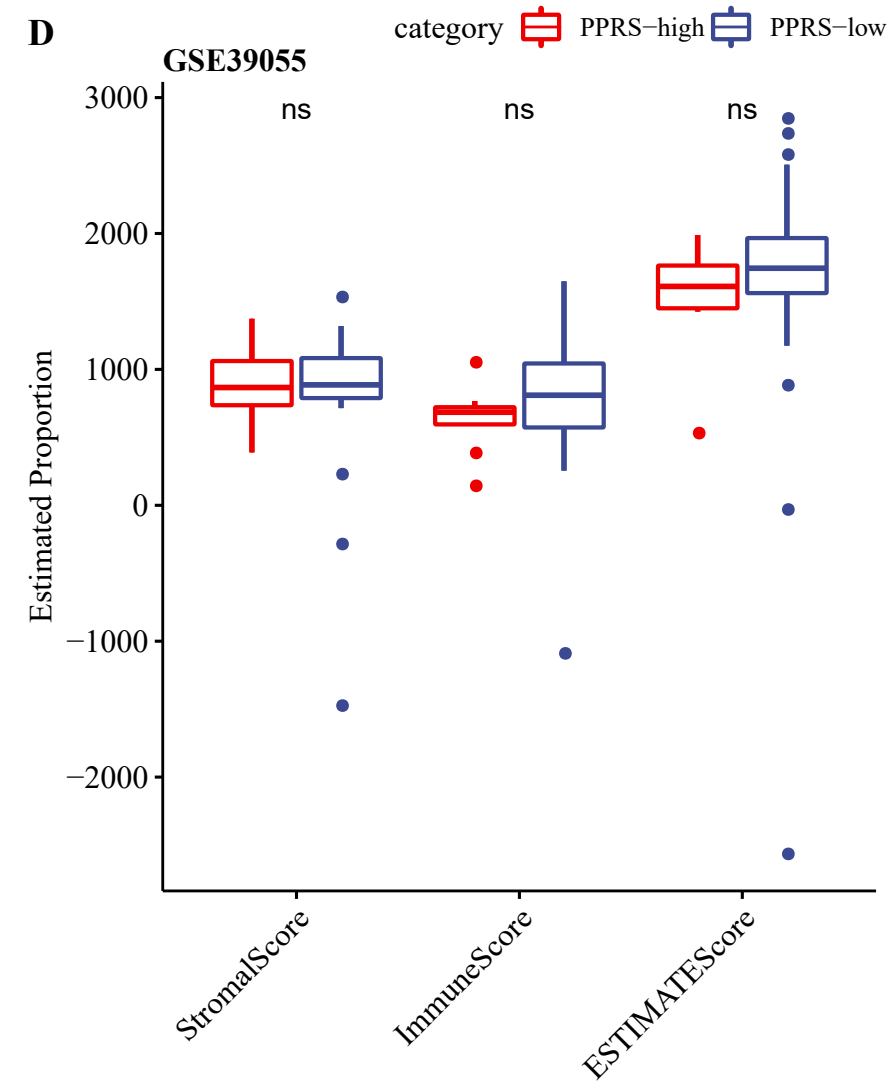

Supplement: Supplementary Materials — Supplementary Figure S1. KEGG and GO function analysis on genes within the purple module. (A) The top 10 enriched KEGG pathways. (B–D) The top 10 enriched GO terms of molecular function, cellular component, and biological process. Supplementary Figure S2. Identifying prognostic genes related to pyroptosis and constructing a prognostic model. (A) Identifying genes in the purple module was significantly associated with prognosis by univariate Cox regression analysis. Log-rank test was conducted. (B–C) LASSO Cox regression analysis for decreasing the number of genes. The dotted red line indicates the optimal lambda value of 0.1395. (D) The LASSO coefficients of five prognostic genes. Supplementary Figure S3. Comparison of TME between high- and low-PPRS groups in TARGET-OS dataset. (A) The proportion of 22 immune cells in two groups. Student's t-test was conducted. (B) Comparison of the stromal score, immune score, and ESTIMATE score between high- and low-PPRS groups. Student's t-test was conducted. (C) Pearson correlation analysis between PPRS score and enrichment of immune cells. Blue and red indicate negative and positive correlations, respectively. ns, not significant. ∗P < 0.05, ∗∗P < 0.01, and ∗∗∗P < 0.001. Supplementary Figure S4. Comparison of TME in GSE21257 (A-B) and GSE39055 (C-D) datasets. ns, not significant. ∗P < 0.05, ∗∗P < 0.01, and ∗∗∗P < 0.001. Supplementary Figure S5. (A) Expression of immune checkpoints in high- and low-PPRS groups. (B) Enrichment of immunosuppressive cells (MDSC, CAF, and M2 TAM), T cell exclusion, T cell dysfunction, and TIDE score in high- and low-PPRS groups. Supplementary Figure S6. (A) Expression of immune checkpoints in high- and low-PPRS groups. (B) Enrichment of immunosuppressive cells (MDSC, CAF, and M2 TAM), T cell exclusion, T cell dysfunction, and TIDE score in high- and low-PPRS groups. Supplementary Figure S7. The estimated IC50 of four chemotherapeutic drugs in TARGET-OS (A), GSE21257 (B), and GSE39055 (C) datasets. [file 1317990.f1.zip › Supplementary Figure S5 (1).pdf]

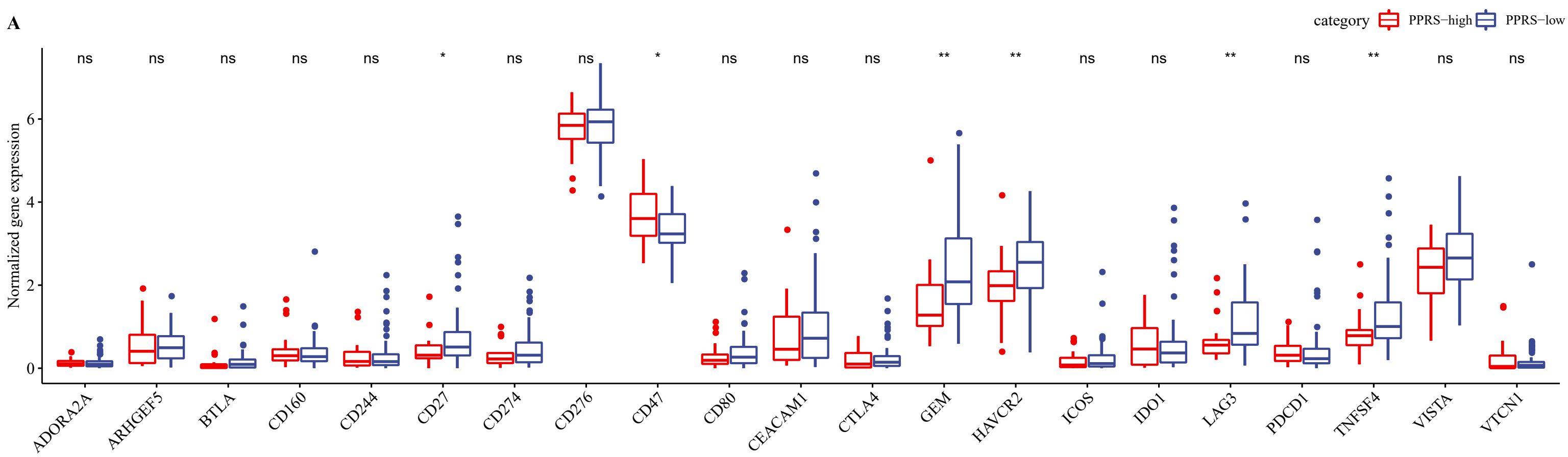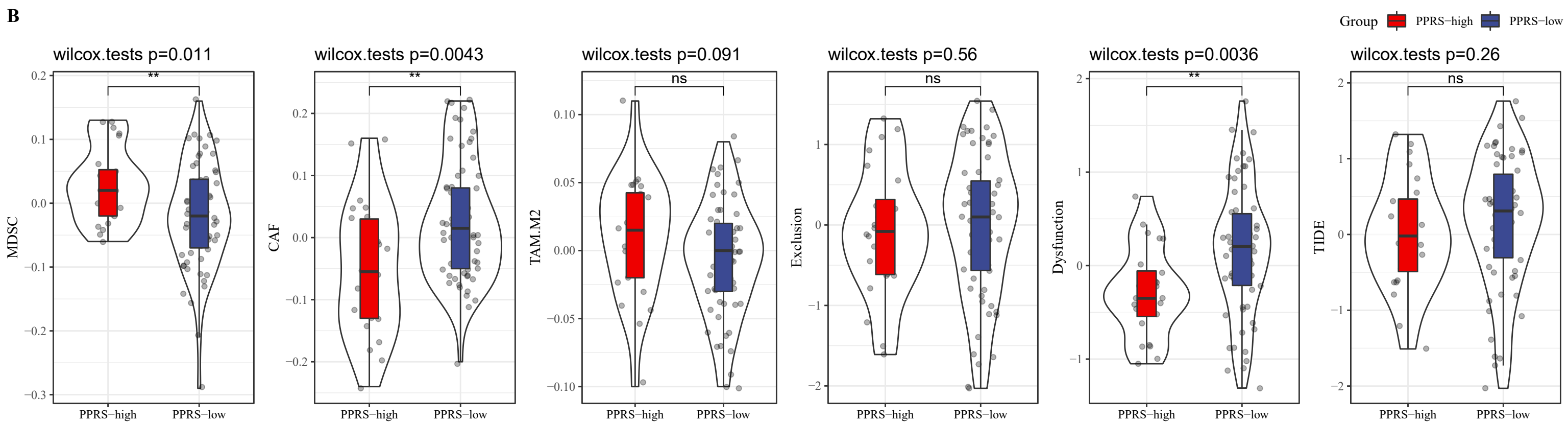

Supplement: Supplementary Materials — Supplementary Figure S1. KEGG and GO function analysis on genes within the purple module. (A) The top 10 enriched KEGG pathways. (B–D) The top 10 enriched GO terms of molecular function, cellular component, and biological process. Supplementary Figure S2. Identifying prognostic genes related to pyroptosis and constructing a prognostic model. (A) Identifying genes in the purple module was significantly associated with prognosis by univariate Cox regression analysis. Log-rank test was conducted. (B–C) LASSO Cox regression analysis for decreasing the number of genes. The dotted red line indicates the optimal lambda value of 0.1395. (D) The LASSO coefficients of five prognostic genes. Supplementary Figure S3. Comparison of TME between high- and low-PPRS groups in TARGET-OS dataset. (A) The proportion of 22 immune cells in two groups. Student's t-test was conducted. (B) Comparison of the stromal score, immune score, and ESTIMATE score between high- and low-PPRS groups. Student's t-test was conducted. (C) Pearson correlation analysis between PPRS score and enrichment of immune cells. Blue and red indicate negative and positive correlations, respectively. ns, not significant. ∗P < 0.05, ∗∗P < 0.01, and ∗∗∗P < 0.001. Supplementary Figure S4. Comparison of TME in GSE21257 (A-B) and GSE39055 (C-D) datasets. ns, not significant. ∗P < 0.05, ∗∗P < 0.01, and ∗∗∗P < 0.001. Supplementary Figure S5. (A) Expression of immune checkpoints in high- and low-PPRS groups. (B) Enrichment of immunosuppressive cells (MDSC, CAF, and M2 TAM), T cell exclusion, T cell dysfunction, and TIDE score in high- and low-PPRS groups. Supplementary Figure S6. (A) Expression of immune checkpoints in high- and low-PPRS groups. (B) Enrichment of immunosuppressive cells (MDSC, CAF, and M2 TAM), T cell exclusion, T cell dysfunction, and TIDE score in high- and low-PPRS groups. Supplementary Figure S7. The estimated IC50 of four chemotherapeutic drugs in TARGET-OS (A), GSE21257 (B), and GSE39055 (C) datasets. [file 1317990.f1.zip › Supplementary Figure S6.pdf]
